# Stimulator of interferon genes agonist augmented antitumor immunity of osimertinib in *Egfr*‐mutated lung cancer

**DOI:** 10.1002/1878-0261.70264

**Published:** 2026-05-21

**Authors:** Jun Nishimura, Tadahiro Kuribayashi, Johannes Brägelmann, Sachi Okawa, Masataka Taoka, Shunta Mori, Tomoka Nishimura, Takaaki Tanaka, Go Makimoto, Kiichiro Ninomiya, Kammei Rai, Eiki Ichihara, Ryohei Katayama, Katsuyuki Hotta, Masahiro Tabata, Yosuke Togashi, Yoshinobu Maeda, Martin L. Sos, Katsuyuki Kiura, Kadoaki Ohashi

**Affiliations:** ^1^ Department of Hematology, Oncology and Respiratory Medicine Okayama University Graduate School of Medicine, Dentistry and Pharmaceutical Sciences Japan; ^2^ Department of Translational Genomics Faculty of Medicine and University Hospital Cologne, University of Cologne Germany; ^3^ Faculty of Medicine and University Hospital Cologne, Mildred Scheel School of Oncology, University of Cologne Germany; ^4^ Center for Molecular Medicine Cologne Faculty of Medicine and University Hospital Cologne Germany; ^5^ Department of Respiratory Medicine Okayama University Hospital Japan; ^6^ Center for Comprehensive Genomic Medicine Okayama University Hospital Japan; ^7^ Center for Innovative Clinical Medicine Okayama University Hospital Japan; ^8^ Center for Clinical Oncology Okayama University Hospital Japan; ^9^ Division of Experimental Chemotherapy, Cancer Chemotherapy Centre Japanese Foundation for Cancer Research Tokyo Japan; ^10^ Department of Computational Biology and Medical Science, Graduate School of Frontier Science, Tokyo The University of Tokyo Japan; ^11^ Department of Translational Oncology German Cancer Research Center (DKFZ) Heidelberg Germany; ^12^ German Cancer Consortium (DKTK), Partner Site Munich, A Partnership Between DKFZ and Ludwig‐Maximilians‐Universität Munich Germany; ^13^ Department of Medicine III LMU University Hospital Munich Germany

**Keywords:** abscopal effect, CD8^+^ T cells, *EGFR* mutation, EGFR tyrosine kinase inhibitor, NK cells, stimulator of interferon genes

## Abstract

*Epidermal growth factor receptor* (*EGFR*)‐mutant non‐small‐cell lung cancers (NSCLCs) lack effective immunotherapy due to a noninflamed tumor microenvironment (TME). We previously reported that EGFR tyrosine‐kinase‐inhibitor (TKI) induced CD8^+^ T‐cell immunity, which was insufficient for tumor eradication. We evaluated the potential of combining EGFR‐TKI with stimulator of interferon genes (STING) agonists in activating a systemic antitumor response. Using a syngeneic mouse model of genetically engineered *Egfr*‐mutant NSCLC, we evaluated the antitumor effects of STING agonist ADU‐S100, alone and combined with osimertinib. Immunohistochemistry and flow cytometry were used to assess the TME. Osimertinib alone enhanced CD8^+^ T‐cell infiltration but not Natural Killer (NK) cell infiltration. ADU‐S100 injection alone modestly suppressed tumor growth with increasing CD8^+^/NK cell infiltration in the TME, but lacked an abscopal effect. Combining ADU‐S100 with osimertinib significantly enhanced the antitumor effects and CD8^+^/NK cell infiltration. Depletion of either CD8^+^ or NK cells reduced the combination effect. Crucially, the combination induced an abscopal effect accompanied by PD‐1^+^/CD8^+^ cell infiltration. Combining osimertinib with a STING agonist augmented innate and adaptive immunity, inducing systemic antitumor responses in *EGFR*‐mutant NSCLC.

## Graphical abstract

Combining osimertinib with the STING agonist ADU‐S100 activates innate and adaptive immunity to overcome the non‐inflamed microenvironment of *Egfr*‐mutant lung cancer. This combination increases NK and CD8^+^ T‐cell infiltration, associated with activation of the STING‐IRF3 pathway and local immunogenic cell death. Crucially, the treatment triggers a systemic abscopal effect primarily mediated by functional CD8^+^ T cells.

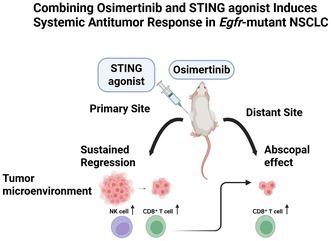

**Abbreviations**
EGFR: epidermal growth factor receptor
FCM: flow cytometry
ICD: immunogenic cell death
ICI: immune checkpoint inhibitor
IHC: immunohistochemistry
NSCLC: non‐small‐cell lung cancer
STING: Stimulator of interferon genes
TKIs: tyrosine kinase inhibitors
TME: tumor microenvironment

## 1 Introduction

Epidermal growth factor receptor (EGFR) gene mutations are detected in up to 60% as driver oncogene mutations in never‐smoking‐related non‐small‐cell lung cancer (NSCLC) [1]. EGFR‐tyrosine kinase inhibitors (TKIs), such as osimertinib, are one of the standard therapies for advanced lung adenocarcinoma harboring *EGFR* mutations; however, resistance and relapse are inevitable within 1–2 years due to genetic or nongenetic mechanisms [2, 3, 4]. While immunotherapy with immune checkpoint inhibitors (ICIs) provides long‐term survival in NSCLC without *EGFR* mutations [5]; NSCLC harboring *EGFR* mutations shows a limited response to ICIs [6].

*EGFR*‐mutated lung cancers generally have a noninflamed tumor microenvironment (TME) with a low number of infiltrating CD8^+^ T cells [7]. Using a genetically engineered mouse model, we previously reported that *Egfr* exon 19 deletion contributes to the formation of a noninflamed TME, and that EGFR‐TKI treatment induced an inflamed TME in lung cancer harboring an *Egfr* mutation [8]. However, CD8^+^ T‐cell‐related antitumor immunity failed to eliminate the cancer cells completely, suggesting that the activation of tumor immunity is insufficient [8]. Furthermore, the clinical development of a combination of EGFR‐TKIs and ICIs has been suspended owing to adverse effects [9]. Therefore, an alternative strategy to enhance the tumor immunity induced by EGFR‐TKIs is warranted.

Stimulator of interferon (IFN) genes (STING), an endoplasmic reticulum resident protein, is associated with the activation of innate or adaptive immunity by promoting the production of type I IFN [10, 11]. Intratumoral administration of a STING agonist leads to the infiltration of natural killer (NK) cells or CD8^+^ T cells into the tumor [12, 13]. Therefore, STING agonists are thought to have the potential to induce and enhance antitumor immunity. Currently, combinations of ICIs and STING agonists, such as ADU‐S100, have been evaluated in clinical trials [14]. However, the role of STING agonists in augmenting the antitumor immunity induced by EGFR‐TKIs in *EGFR*‐mutated lung cancers remains unknown. Consequently, we aimed to investigate the role of the STING agonist, ADU‐S100, in a syngeneic lung cancer mouse model harboring *Egfr* exon 19 deletion.

## 2 Materials and methods

### 2.1 Reagents and antibodies

For *in vivo* experiments, osimertinib was purchased from Selleck (Houston, TX, USA). ADU‐S100 disodium salt (cat. no. HY‐12885A) was purchased from MedChemExpress (Monmouth Junction, NJ, USA). Anti‐CD8α antibody (clone 53‐6.7) was purchased from BioLegend (San Diego, CA, USA). Anti‐mouse NK1.1 (PK136) was purchased from Bio X Cell (Lebanon, NH, USA).

The following primary antibodies for immunohistochemistry (IHC) were used: anti‐CD8α (EPR21769), anti‐EGFR (EP38Y), anti‐interferon regulatory factor 3 (IRF3) (EPR2418Y), and anti‐Calreticulin (CRT) (EPR3924) were purchased from Abcam (Cambridge, UK). NK1.1 (E6Y9G), CD11c (D1V9Y), Granzyme B (E5V2L), and HMGB1 (D3E5) were purchased from Cell Signaling Technology (Danvers, MA, USA). Phosphorylated (p‐)IRF3 (Ser396) was purchased from Proteintech (Rosemont, IL, USA).

An EnVision+ System‐labeled polymer‐horseradish peroxidase anti‐rabbit antibody (cat. no. K4002; Dako, Glostrup, Denmark) was used as the secondary antibody for IHC. The Liquid DAB+ Substrate Chromogen System was used for 3,3′‐diaminobenzidine (DAB) staining (cat. no. K3468; Dako).

Flow cytometry (FCM) antibodies anti‐CD8α (53‐6.7), anti‐CD4 (GK1.5), anti‐PD‐1 (29F.1A12), rat IgG2a (RTK2758), anti‐NK1.1 (S17016D), anti‐CD69 (H1.2F3), Armenian Hamster IgG Isotype Ctrl Antibody (HTK888), anti‐H‐2Db (KH95), and mouse IgG2b (MPC‐11) were purchased from BioLegend. CD3 (17A2), anti‐H‐2Kb (AF6‐88.5.5.3), and mouse IgG2a (eBM2a) were purchased from Thermo Fisher Scientific (Waltham, MA, USA).

### 2.2 Cell culture

The PC‐9 (*EGFR Exon 19 del E746_A750*) cell line (RRID:CVCL_B260) was purchased from the European Collection of Authenticated Cell Cultures (Salisbury, UK) and the H1975 (*EGFR L858R + T790M*) cell line (RRID:CVCL_1511) was purchased from the American Type Culture Collection (Rockville, MD, USA). Cell identity was confirmed with short tandem repeat polymorphism analysis. All cell lines were cultured with a maximum of 20 passages. The cell lines were verified as mycoplasma free before starting the experiments (cat. no. 25235; e‐Myco Mycoplasma PCR Detection Kit (Ver2.0), iNtRON Biotechnology, Inc., Seongnam, South Korea). The cells were cultured in Roswell Park Memorial Institute‐1640 medium (cat. no. R8758; Sigma‐Aldrich, Tokyo, Japan) supplemented with 10% heat‐inactivated fetal bovine serum (cat. no. 10735078001; Sigma‐Aldrich) and 1% of penicillin/streptomycin (cat. no. 1514022; Thermo Fisher) in a humidified tissue culture incubator at 37 °C under 5% CO_2_.

The murine *Egfr*‐mutant lung cancer cell line (mDEL) was derived from the syngeneic tumor model used in this study [15]. mDEL cells were cultured in a modified medium designated as 50% ESC + Y AA medium, which was prepared by mixing equal volumes of two components: ESC + Y AA (100%) medium consisting of StemPro hESC SFM (cat. no. A1000701; Thermo Fisher Scientific) supplemented with Y‐27632 and 1× Antibiotic‐Antimycotic (cat. no. 161‐23181; penicillin–streptomycin–amphotericin B, Wako, Osaka, Japan), and ESC + Y AA (0%) medium serving as a StemPro‐free base consisting of DMEM/Ham's F‐12 (cat. no. 042‐30555; Wako), 1% bovine serum albumin [cat. no. A10008‐01; 25% BSA solution, supplied with StemPro hESC SFM kit (cat. no. A1000701)], 4.8 ng·mL^−1^ recombinant human FGF‐basic (cat. no. 100‐18B; PeproTech, Rocky Hill, NJ, USA), 60 μm 2‐mercaptoethanol (cat. no. 21985‐023; Thermo Fisher Scientific), 6 μm Y‐27632 (cat. no. HY‐10071; MedChemExpress), and 1× penicillin–streptomycin–amphotericin B (cat. no. 161‐23181; Wako).

### 2.3 Crystal violet assay

PC9 and H1975 cells were seeded in six‐well plates at a density of 15 000 cells/well, and mDEL cells were seeded in six‐well plates at a density of 150 000 cells/well. Treatment with dimethyl sulfoxide (DMSO), osimertinib (1 μmol·L^−1^ for PC‐9 and H1975; 0.1 μmol·L^−1^ for mDEL), and ADU‐S100 (10 μmol·L^−1^) was initiated the following day. Cells were stained 4 days after treatment initiation.

After fixation in 10% formalin for 10 min, the cells were stained for 10 min with crystal violet solution (cat. no. 1092180500; Sigma‐Aldrich) and then washed with H_2_O. The plates were then dried overnight.

### 2.4 Syngeneic *Egfr*‐mutant lung cancer mouse model

This study was approved by the Animal Care and Use Committee of the Okayama University, Okayama, Japan (OKU‐2020228). Female C57BL/6J mice aged 6–8 weeks were purchased from Jackson Laboratory, Japan. All mice were provided with sterilized food and water, housed in a barrier facility, and maintained at an air‐conditioned temperature of 22 ± 2 °C, with constant humidity under a 12‐h/12‐h light/dark cycle. The mice were monitored twice‐weekly. Subcutaneous tumors harboring *Egfr exon 19 deletions* were passaged using C57BL/6J mice, as previously described [16, 17]. For this study, the subcutaneous *Egfr*‐mutated lung tumors were minced and dissociated into single‐cell suspensions using a mouse Tumor Dissociation Kit (cat. no. 130‐096‐730; Miltenyi Biotec), and red blood cells were removed from the suspensions using a Red Blood Cell Lysis Solution (cat. no. 130‐094‐183; Miltenyi Biotec). A suspension solution of 50–120 × 10^4^ tumor cells in 0.10 mL of phosphate‐buffered saline (PBS) mixed with 0.10 mL of Matrigel matrix (cat. no. 356237; Corning) was prepared (for each tumor per mouse) and injected into one or both flanks of the mice.

When the average volume of transplanted tumors reached approximately 200–300 mm^3^ after the subcutaneous transplantation, the mice were randomly assigned into the vehicle control or monotherapies or combinations therapy groups: For monotherapies‐PBS (100 μL, by intratumoral administration [i.t.], Day 1), osimertinib (15 mg·kg^−1^·day^−1^, by oral gavage [p.o.], 7 days/week for 14 days) and ADU‐S100 (50 μg, by i.t., Day 1) and for combinations—combination osimertinib (15 mg·kg^−1^·day^−1^, p.o., 7 days/week for 3 or 14 days) and ADU‐S100 (50 μg, i.t., day 1). Tumors were harvested for analysis at Day‐ 0, 4, 14, and Day 7 (3 days after the last osimertinib treatment).

For the continuous depletion of target immune cell populations prior to and during treatment, anti‐mouse CD8a (250 μg/injection, intraperitoneally [i.p.]) or anti‐mouse NK1.1 (300 μg/injection, i.p.) antibodies were administered on Day 0 (prior to treatment initiation), and subsequently on Days 3, 10, and 17. Depletion efficiency was evaluated by immunohistochemical analysis of spleens harvested on Days 4 and 14 (Fig. S4B). When examining the abscopal effect, anti‐mouse CD8^+^ and NK1.1^+^ antibodies were each administered at the same dose on Days 4, 7, 10, and 13 after the start of treatment. The mice were kept in the study until tumors reached 2000 mm^3^.

### 2.5 Immunohistochemistry

IHC was conducted using tumors from the syngeneic *Egfr*‐mutant lung cancer mouse model, as described previously [18, 19]. Formalin‐fixed, paraffin‐embedded tissues were cut to a thickness of 5 μm. Where multiple markers were compared within the same tumor regions, serial sections were used. The sections were placed on glass slides, and deparaffinized as follows: rinsed thrice in Hemo‐De (cat. no. CS‐1001‐4, FALMA; Tokyo, Japan) for 5 min and then soaked in 99.5% ethanol (cat. no. 09‐0770‐5; Sigma‐Aldrich) for 2 min, 95% ethanol for 2 min, 70% ethanol for 2 min, and pure water for 3 min. The slides were incubated in pure water containing 1 mmol·L^−1^ ethylenediaminetetraacetic acid (EDTA) (cat. no. 15575‐020; Invitrogen) or Tris‐EDTA (cat. no. PR30002; Proteintech) for 10 min in a 95 °C Pascal (cat. no. S2800; Dako) and soaked in 0.3% hydrogen peroxide (cat. no. 18412; Santoku Chemical, Tokyo, Japan) with methanol (cat. no. 21915‐93; Nacalai Tesque, Kyoto, Japan) as a solvent to inactivate endogenous peroxidases for 5 min. The slides were rinsed with Tris‐buffered saline (pure water containing 20 mmol·L^−1^ trizma base [cat. no: T1503; Sigma‐Aldrich]) and 137 mmol·L^−1^ NaCl (cat. no: 28‐2270‐5; Sigma‐Aldrich) and adjusted to pH 7.0 with HCl (cat. no. 37338‐15; Nacalai Tesque) with 0.10% polyoxyethylene sorbitan monolaurate (cat. no. 35624‐15; Nacalai Tesque), and the sections were incubated with 240 μL of wash buffer (cat. no. S3006; Dako) and 30 μL of goat serum (cat. no. 01‐6201; Invitrogen) for 60 min at approximately 25 °C. The following antibodies were used: anti‐CD8a (EPR21769, 1/2000; Abcam), anti‐NK1.1 (E6Y9G, 1/800; Cell Signaling Technology), anti‐CD11c (D1V9Y, 1/350; Cell Signaling Technology), anti‐EGFR (EP38Y, 1/100; Abcam), anti‐IRF3 (EPR2418Y, 1/500; Abcam), anti‐p‐IRF3 (Ser396, 1/2000; Proteintech), Granzyme B (E5V2L, 1/100; Cell Signaling Technology), anti‐CRT (EPR3924, 1/200; Abcam), and anti‐HMGB1(D3E5, 1/200; Cell Signaling Technology).

Dako wash buffer was used as the diluent (cat. no. S3006; Dako). The sections were incubated overnight at 4 °C with the above primary antibodies, followed by incubation with a secondary antibody (1/1 undiluted, cat. no. K4003; Dako) for 20 min at room temperature. DAB staining was performed using a Liquid DAB+ Substrate Chromogen System (cat. no. K3468; Dako) and counterstained with hematoxylin (cat. no: 30002; MUTO Pure Chemicals, Tokyo, Japan).

The samples were evaluated under a microscope (cat. no. BZ8100; Keyence, Osaka, Japan). The percentage of the DAB+ area was measured using the imagej software (version 1.53t; National Institutes of Health, Bethesda, MD, USA).

### 2.6 Flow cytometry

Tumor tissues from the syngeneic *Egfr*‐mutant lung cancer mouse model were dissected from the mice and dissociated into single‐cell suspensions, as described above, and red blood cells were removed using a red blood cell lysis solution (cat. no. 130‐094‐183; Miltenyi Biotec, Bergisch Gladbach, Germany). Cells, including tumor‐infiltrating lymphocytes and tumor cells, were stained with the fluorescently labeled antibodies indicated above and subjected to FCM analysis. Briefly, the cells were washed with fluorescence‐activated cell sorting (FACS) staining buffer containing 2 mmol·L^−1^ EDTA (cat. no. 15575020; Thermo Fisher Scientific) and 0.50% (w/v) bovine serum albumin (cat. no. 10735078001; Sigma‐Aldrich) in PBS (pure water containing 0.02% KCl [cat. no. 24‐3290; Sigma‐Aldrich], 0.80% NaCl [cat. no. 28‐2270‐5; Sigma‐Aldrich], 0.115% Na_2_HPO_4_ [cat. no. 28‐3750‐5; Sigma‐Aldrich], 0.02% KH_2_PO_4_ [cat. no. 169‐04245; Sigma‐Aldrich]) and incubated with monoclonal antibodies against surface markers for 30 min at 4 °C in the FACS staining buffer. DAPI (cat. no. 422801; BioLegend) was used to assess cell viability.

The samples were acquired using a MACSQuant flow cytometer (Miltenyi Biotec), and the data were analyzed using the FlowJo software (version 10; TreeStar Inc., Ashland, OR, USA). To assess major histocompatibility complex (MHC) class I expression and its intratumoral heterogeneity, the mean fluorescence intensity (MFI) and the robust coefficient of variation (rCV) were calculated.

### 2.7 RNA sequencing, cell‐type deconvolution, and gene set enrichment analysis

RNA sequencing and analysis were performed as described previously [20, 21]. Total RNA was extracted from fresh‐frozen mouse tumors and 3′ UTR mRNA sequencing libraries were prepared using Lexogen QuantSeq kits according to the manufacturer's instructions. Raw sequencing reads were aligned to the mouse reference genome with the STAR aligner (v2.7.0e), and expression levels were quantified using RSEM v1.3.1. Cell‐type deconvolution was performed using the murine Microenvironment Cell Population (mMCP) counter method [22]. Gene Set Enrichment Analysis (GSEA) was conducted on the RNA‐sequencing data after pre‐ranking of genes by significance and fold‐change between conditions [20].

### 2.8 Quantitative real‐time polymerase chain reaction (qPCR) assay

Total RNA from tumor tissues harvested on Day 0, 4, and 14 was extracted using the Quick‐DNA/RNA Miniprep Plus Kit (cat. no. D7003; Zymo Research, Irvine, CA, USA), and reverse transcription was performed using SuperScript II Reverse Transcriptase (cat. no. 18064014; Thermo Fisher Scientific) according to the manufacturers' protocols. Quantitative real‐time PCR was performed using TaqMan™ Gene Expression Assays with TaqMan™ Fast Advanced Master Mix (cat. no. 4444557; Thermo Fisher Scientific) on a LightCycler 96 (Roche Diagnostics, Mannheim, Germany). The thermal profile was 50 °C for 2 min, 95 °C for 20 s, followed by 40 cycles of 95 °C for 3 s and 60 °C for 30 s. The expression levels of *Cd8a* (Assay ID: Mm01182107_g1), *Ifng* (Assay ID: Mm01168134_m1), *Cxcl9* (Assay ID: Mm00434946_m1), and *Gzmb* (Assay ID: Mm00442837_m1) were normalized to *Gapdh* (Assay ID: Mm99999915_g1), and relative expression was calculated from mean quantification cycle (*C*_q_) values.

### 2.9 Statistical analyses

Statistical analyses were performed using graphpad prism 9.4.0 (GraphPad Software, San Diego, CA, USA). Two‐sided Student's *t*‐tests were used to compare the means of data between two groups, and one‐way analysis of variance with post hoc Tukey's test was used for comparisons among multiple independent groups, unless otherwise specified. Statistical significance was set at *P* < 0.05. The combination effect *in vivo* was evaluated using the Bliss independence model. The expected fractional tumor volume (FTV) was calculated using the following formula: FTV_(expected)_ = FTV_(osimertinib)_ × FTV_(ADU‐S100)_. FTV represents the ratio of the mean tumor volume of each treatment group to that of the vehicle control. Synergy was defined as an observed FTV lower than the expected FTV (i.e., FTV_(observed)_ < FTV_(expected)_).

## 3 Results

### 3.1 Induction of NK cells with osimertinib was limited in *Egfr*‐mutant lung cancer

**Fig. 1:**
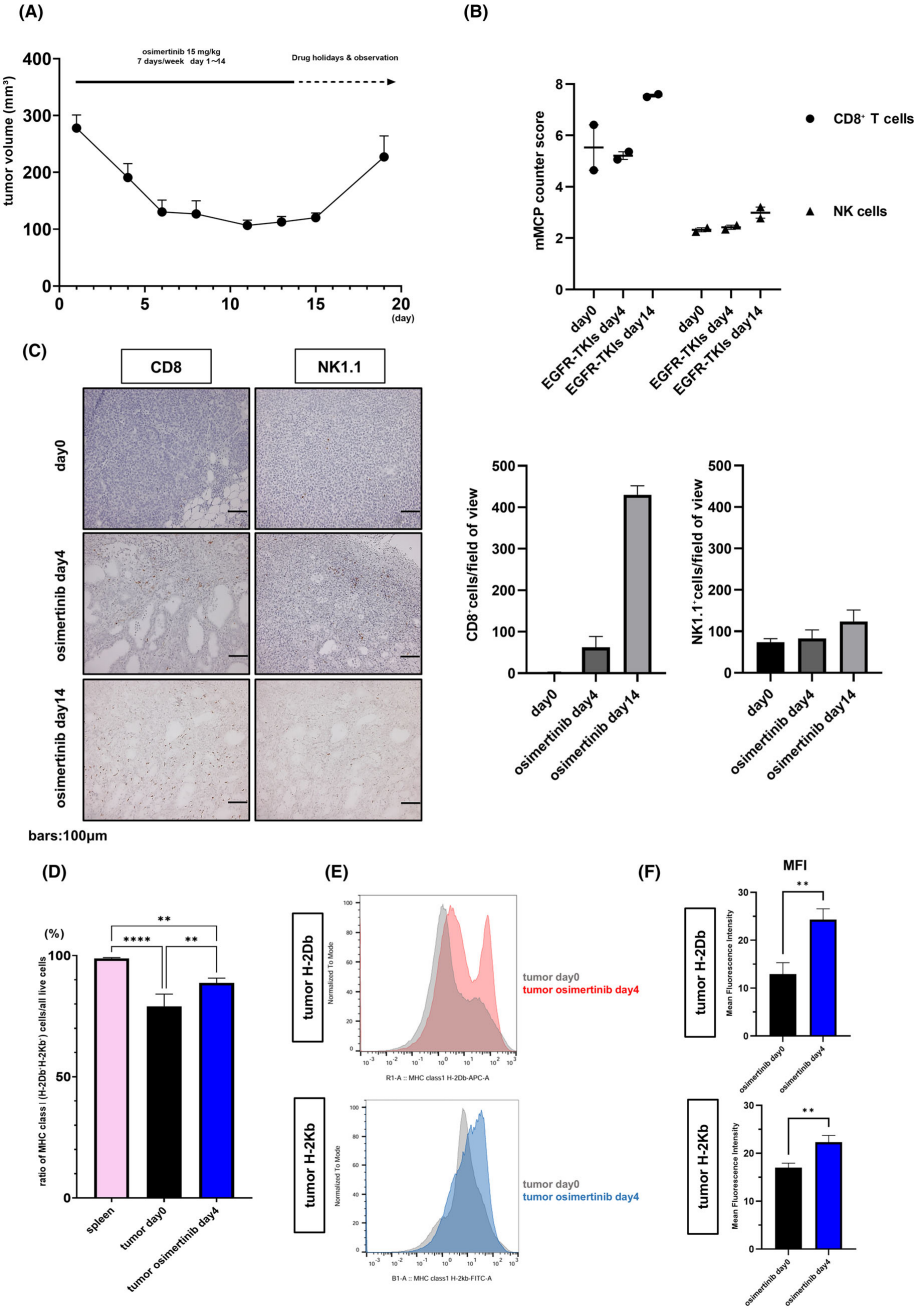
Induction of antitumor immunity by osimertinib and major histocompatibility complex (MHC) class I expression in *Egfr*‐mutant lung cancer. (A) Tumor growth in the *Egfr*‐mutant lung cancer model treated with osimertinib (15 mg·kg^−1^·day^−1^, oral gavage [p.o.], 14 days), (*n* = 8 tumors per group, 4 mice per group). Error bars represent the standard error. Data shown are representative of three independent experiments with similar results. (B) Classification of cell types by RNA sequencing over time in *Egfr*‐mutant lung cancer mouse model treated with osimertinib or gefitinib (one tumor per treatment, *n* = 2 tumors total). (C) Representative images of CD8 and NK 1.1 immunohistochemistry staining on tumors treated with osimertinib (15 mg·kg^−1^·day^−1^, p.o.) after 4 or 14 days. The CD8^+^ and NK1.1^+^ cells were quantified using the imagej software. Error bars represent the standard error (*n* = 5 fields of view per group). Scale bars: 100 μm. Data shown are representative of two independent experiments with similar results. (D) MHC class I expression in the tumor. Ratio of MHC class I (H‐2Db^+^ and H‐2Kb^+^) cells in all live cells *n* = 3 spleens, *n* = 6 tumors per group, 3 mice per group. Error bars represent the standard error. **P < 0.01, ****P < 0.0001, one‐way ANOVA with the post hoc Tukey test. Data shown are representative of two independent experiments with similar results. (E) Representative flow cytometry histograms showing MHC class I proteins (H‐2Db and H‐2Kb) expression in dissociated tumor cells. (F) Mean fluorescence intensity (MFI) (Day 0: *n* = 12 per group, Day 4: *n* = 10 per group) for H‐2Db and H‐2Kb is shown. Error bars represent the standard error. **P < 0.01, Student's *t*‐test. Data are the integrated results of two independent experiments.

Using a syngeneic *Egfr*‐mutant lung cancer mouse model, we first evaluated the antitumor effect of osimertinib. When the average tumor volume reached approximately 200–300 mm^3^, the tumor‐bearing mice were treated with osimertinib (15 mg·kg^−1^ p.o., 7 days/week) for 14 days, followed by 5 days of observation without treatment (Fig. 1A). Consistent with our previous study [8, 23], the third‐generation EGFR‐TKI, osimertinib, showed a tumor inhibitory effect on tumor growth in a lung cancer model *in vivo*; however, the tumors regrew during the observation period. We next assessed changes in immune cells in tumors treated with EGFR‐TKIs. RNA sequencing was performed on tumors without treatment (Day 0) and on those treated with osimertinib (15 mg·kg^−1^) or gefitinib (50 mg·kg^−1^) for 4 or 14 days. To estimate the composition of immune cells in the TME, mMCP counter analysis was applied to the RNA‐sequencing data. CD8^+^ T‐cell‐related genes showed a trend toward increase in tumors treated with EGFR‐TKIs for 14 days compared with untreated tumors or those treated with EGFR‐TKIs for 4 days (Fig. 1B). GSEA was performed to evaluate pathway enrichment in untreated mouse tumors versus those treated EGFR‐TKI for 14 days. The Hallmark IFNγ response signature was identified as the most upregulated in the treated tumors with a normalized enrichment score of 9.96 (adj. *P* < 0.0001; Fig. S1A). To validate these findings in independent biological replicates (*n* = 4 per group), we performed qPCR. Consistent with the RNA‐sequencing data, qPCR analysis demonstrated a significant upregulation of *Cd8a*, *Ifng*, and *Gzmb* at Day 14 compared to Day 0, while *Cxcl9* showed a nonsignificant increasing trend (Fig. S1B). However, the increase in NK cell‐related genes, which have antitumor effects against tumors resistant to cytotoxic T lymphocyte [13], was modest in tumors treated with EGFR‐TKIs (Fig. 1B). IHC analysis confirmed an increase in the number of CD8^+^ cells in tumors treated with osimertinib for 14 days compared with untreated tumors and those treated with osimertinib for 4 days. While the number of NK1.1^+^ cells showed a slight increase, these trends were consistent across replicates (Fig. 1C). The infiltration of NK cells into the tumor microenvironment was limited by osimertinib treatment, suggesting that enhanced NK cell recruitment may augment antitumor efficacy [13].

Given the enhanced antitumor effects of NK cells upon recognizing abnormal MHC class I expression, we next assessed MHC class I expression in our *Egfr*‐mutant lung cancer mouse model. MHC class I positivity was defined as co‐expression of both H‐2Db and H‐2Kb in all live cells. As expected, MHC class I expression in the tumors was significantly lower than that in the spleen, used as a positive control. Consistent with our previous study [8], the expression of MHC class I molecules increased in tumors treated with EGFR‐TKIs (Fig. 1D, Fig. S2A). Flow cytometry histograms demonstrated a rightward shift of the cell population for both H‐2Kb and H‐2Db on Day 4 compared to Day 0 (Fig. 1E, Fig. S2A). Consistent with this, the MFI for both subclasses was significantly increased (*P* < 0.01) (Fig. 1F). To quantitatively assess intratumoral heterogeneity, we evaluated the rCV. The rCV values for both H‐2Db and H‐2Kb showed no significant change between Day 0 and Day 4 (Fig. S2B), indicating that the distributional spread did not broaden, inconsistent with the selective outgrowth of a minor high‐expressing subpopulation. Furthermore, this overall upregulation of MHC class I expression coincided with a significant increase in Granzyme B^+^ cells (Fig. S2C), suggesting that this MHC class I upregulation is associated with an enhanced cytotoxic immune response. However, despite this upregulation, MHC class I expression remained lower in tumors treated with EGFR‐TKI compared to the spleen cells (Fig. 1D). This finding provides a rationale for the hypothesis that NK cells may retain the potential to recognize and target *Egfr*‐mutant lung cancer based on the missing‐self hypothesis [24].

### 3.2 ADU‐S100 induced NK and CD8 ^+^ cells into the tumor and augmented antitumor effect *in vivo*

**Fig. 2:**
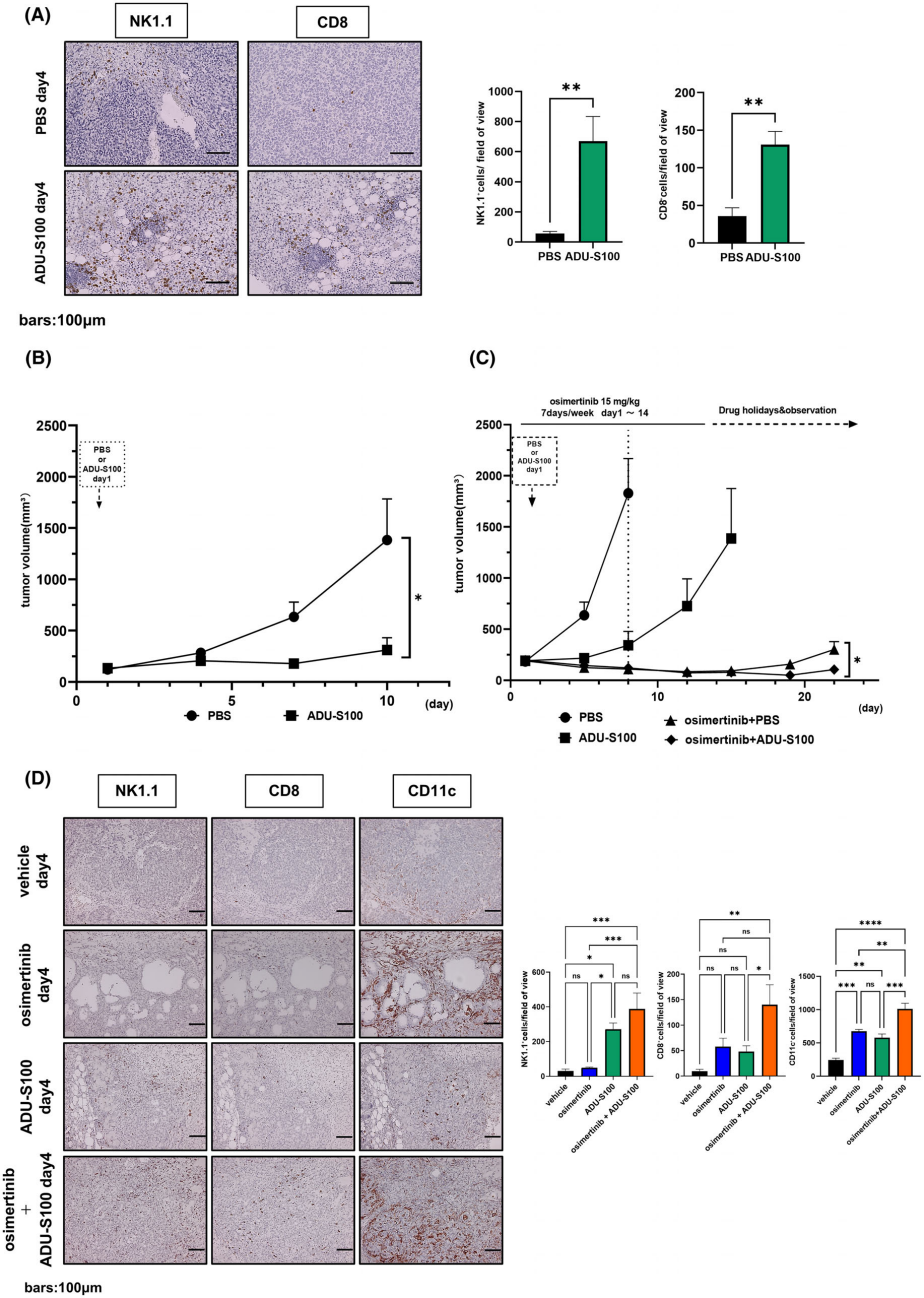
Antitumor effect of ADU‐S100 in *Egfr*‐mutant lung cancer mouse model. (A) Representative images of NK1.1 and CD8 immunohistochemistry (IHC) staining on tumors from *Egfr*‐mutant mice treated with phosphate‐buffered saline (PBS) (100 μL, intratumoral administration [i.t.], Day 1 as vehicle) or ADU‐S100 (50 μg, i.t., Day 1) after 4 days. The NK1.1^+^ and CD8^+^ cells were quantified using the imagej software. Error bars represent the standard error (*n* = 5 fields of view per group). Scale bars: 100 μm. **P < 0.01, Student's *t*‐test. Data shown are representative of three independent experiments with similar results. (B) The effect of ADU‐S100. Tumor growth in the *Egfr*‐mutant lung cancer mouse model treated with PBS (100 μL, i.t., Day 1 as vehicle) or ADU‐S100 (50 μg, i.t., Day 1) (*n* = 3 tumors per group, 3 mice per group). Error bars represent the standard error. *P < 0.05, Student's *t*‐test. Data shown are representative of three independent experiments with similar results. (C) Combination effect of osimertinib and ADU‐S100. Tumor growth in the *Egfr*‐mutant lung cancer model treated with PBS (100 μL, i.t., Day 1 vehicle; *n* = 4 tumors from 2 mice per group), osimertinib (15 mg·kg^−1^·day^−1^, oral gavage [p.o.], 14 days; *n* = 6 tumors from 3 mice per group), ADU‐S100 (50 μg, i.t., Day 1; *n* = 6 tumors from 3 mice per group), or combination of osimertinib (15 mg·kg^−1^·day^−1^, p.o., 14 days; *n* = 6 tumors from 3 mice per group) and ADU‐S100 (50 μg, i.t., Day 1). A vertical dashed line at Day 8 indicates the time point used for Bliss independence model analysis. Error bars represent the standard error. *P < 0.05, Student's *t*‐test. Data shown are representative of two independent experiments with similar results. (D) Representative images of NK1.1, CD8, and CD11c IHC staining on *Egfr*‐mutant lung cancer tumors from mice treated with PBS (100 μL, i.t., Day 1 as vehicle), osimertinib (15 mg·kg^−1^·day^−1^, p.o.), ADU‐S100 (50 μg, i.t., Day 1), and combination of osimertinib (15 mg·kg^−1^·day^−1^, p.o.) and ADU‐S100 (50 μg, i.t., Day 1) for 4 days. The NK1.1^+^, CD8^+^ and CD11c^+^ cells were quantified using imagej software. Error bars represent the standard error (*n* = 5 fields of view per group). Scale bars: 100 μm. ns = not significant, *P < 0.05, **P < 0.01, ***P < 0.001, ****P < 0.0001, one‐way analysis of variance (ANOVA) with the post hoc Tukey test. Data shown are representative of three independent experiments with similar results. Serial sections of the same tumor tissue were used for the various immunostainings shown in this panel.

Given that the STING agonist ADU‐S100 is known to induce NK or CD8^+^ T‐cell infiltration into tumors [13], we assessed the immune cell infiltration in tumors 4 days postinjection of PBS or ADU‐S100 in the *Egfr*‐mutant lung cancer model. As expected, ADU‐S100 significantly increased the infiltration of NK1.1^+^ or CD8^+^ cells into the tumors compared to PBS (Fig. 2A). Additionally, ADU‐S100 demonstrated significantly greater antitumor activity than PBS, albeit the effect was transient (Fig. 2B,C).

Given these immune‐activating effects of ADU‐S100, we next investigated whether its addition to osimertinib could confer more durable tumor control, applying the Bliss independence model to quantify the combination effect during the early treatment phase (Day 8; Fig. 2C). The expected FTV was calculated as 0.0112 (FTV: 0.0599 for osimertinib and 0.1873 for ADU‐S100), whereas the observed FTV was 0.0664, indicating no synergistic tumor suppression at this time point. This was consistent with the comparable tumor shrinkage observed between osimertinib monotherapy and the combination therapy at Day 8 (*P* = 0.9999, Tukey's *post hoc* test). During the 14‐day treatment period, both osimertinib monotherapy and the combination of osimertinib and ADU‐S100 showed comparable antitumor activity. However, upon drug withdrawal, tumor regrowth was observed in the osimertinib monotherapy group, whereas the combination treatment group showed sustained tumor inhibition (Fig. 2C). These findings suggest that the primary benefit of the combination therapy lies in the durability of antitumor response, rather than a synergistic direct tumor‐suppressive effect.

To assess the infiltration of NK1.1^+^ and CD8^+^ cells, we also performed IHC in the tumors after 4 days of treatment, as well as in those treated for 14 days followed by a 3‐day drug withdrawal (Day 17). NK1.1^+^ cells did not show an increase with osimertinib monotherapy, whereas a significant increase was observed with ADU‐S100 monotherapy or combination therapy compared to vehicle control or osimertinib monotherapy at Day 4 (Fig. 2D). CD8^+^ cell infiltration showed a similar increase with both osimertinib and ADU‐S100 monotherapies, but the difference was not statistically significant. In contrast, the combination therapy significantly increased CD8^+^ cell infiltration compared to ADU‐S100 monotherapy or vehicle control at Day 4 (Fig. 2D). At Day 17 (3 days after the discontinuation of osimertinib), the significantly increased number of NK1.1^+^ and CD8^+^ cells was maintained in the tumors treated with the combination therapy compared to osimertinib monotherapy (Fig. S3). Given that STING agonists are known to induce dendritic cells [25] and that interplay between NK cells and dendritic cells can mediate antitumor immunity [26], the expression of CD11c, a marker of dendritic cells, was also evaluated. Consistent with increased CD8^+^ cells, CD11c^+^ cells also significantly increased in the tumors treated with osimertinib monotherapy compared to vehicle control. Furthermore, CD11c^+^ cells showed a greater increase in tumors treated with combination therapy compared to osimertinib monotherapy at day 4 and 17 (Fig. 2D, Fig. S3).

### 3.3 ADU‐S100 augmented antitumor immunity of osimertinib in *Egfr*‐mutant tumors

**Fig. 3:**
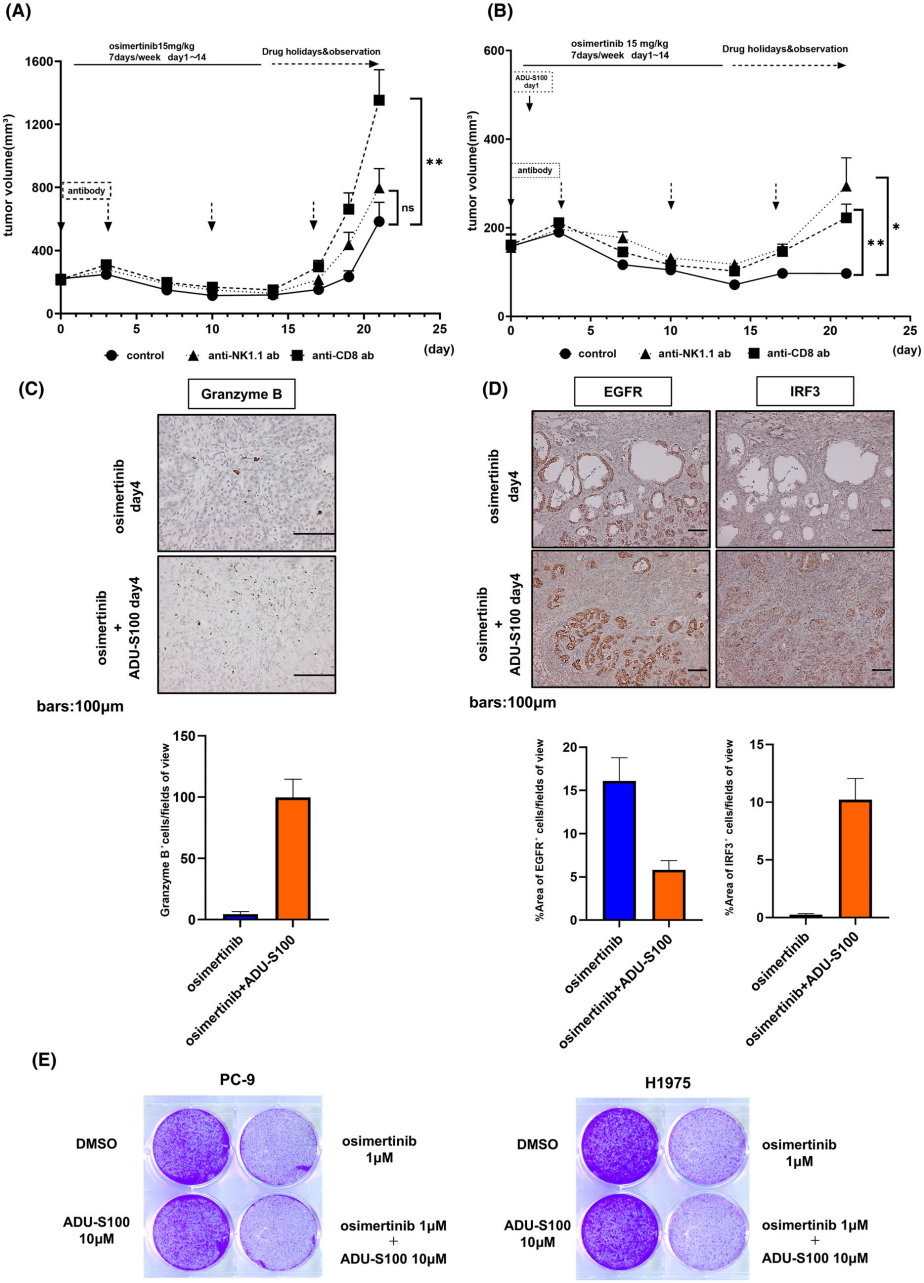
Enhancement of antitumor immunity via CD8^+^ cells and NK1.1^+^ cells in combination therapy. (A) Tumor growth in *Egfr*‐mutant lung cancer mice treated with osimertinib (15 mg·kg^−1^·day^−1^, oral gavage [p.o.], 7 days/week) for 14 days and observed for 7 days with anti‐CD8 or anti‐NK1.1 antibodies. *n* = 6 tumors per group, 3 mice per group. Data shown are representative of two independent experiments with similar results. Error bars represent the standard error. ns = not significant, **P < 0.01, Student's *t*‐test. (B) Tumor growth in mice treated with osimertinib (15 mg·kg^−1^·day^−1^, p.o., 7 days/week) for 14 days and ADU‐S100 (50 μg, intratumoral administration [i.t.]., Day 1) and observed for 7 days with anti‐CD8 or NK1.1 antibodies. *n* = 6 tumors per group, 3 mice per group. Data shown are representative of two independent experiments with similar results. Error bars represent the standard error. *P < 0.05, **P < 0.01, Student's *t*‐test. (C) Representative images of Granzyme B immunohistochemistry (IHC) staining treated with osimertinib (15 mg·kg^−1^·day^−1^, p.o., 7 days/week) or combination of osimertinib (15 mg·kg^−1^·day^−1^, p.o., 7 days/week) and ADU‐S100 (50 μg, i.t., on Day 1). Tumors were harvested on Day 4 after treatment initiation. Granzyme B^+^ cells were quantified using the imagej software. Scale bars: 100 μm. Error bars represent the standard error (*n* = 5 fields of view per group). Data shown are representative of two independent experiments with similar results. (D) Representative images of EGFR and interferon regulatory factor 3 (IRF3) IHC staining treated with osimertinib (15 mg·kg^−1^·day^−1^, p.o.) or combination of osimertinib (15 mg·kg^−1^·day^−1^, p.o.) and ADU‐S100 (50 μg, i.t., on Day 1) Tumors were harvested on Day 4 after treatment initiation. %Area of EGFR^+^ and IRF3^+^ cells was quantified using the imagej software. Scale bars: 100 μm. Error bars represent the standard error (*n* = 5 fields of view per group). Data shown are representative of two independent experiments with similar results. Serial sections of the same tumor tissue were used for the various immunostainings shown in this panel. (E) Crystal violet‐stained cells after drug loading for 4 days. Cancer cells were treated with 0.2% dimethyl sulfoxide, 1 μmol·L^−1^ osimertinib, and/or 10 μmol·L^−1^ ADU‐S100.

Given that ADU‐S100 increased the number of NK1.1^+^ or CD8^+^ cells in the TME and augmented the antitumor effect of osimertinib, we hypothesized that these immune cells contribute to the observed antitumor effect. To ensure that both NK cell and CD8^+^ cell responses were abrogated across the entire therapeutic window, depletion antibodies were initiated prior to treatment (Day 0) and administered continuously throughout the treatment period (Fig. S4A). IHC analysis confirmed the effectiveness of this protocol, demonstrating the sustained depletion of CD8^+^ and NK1.1^+^ cells in the spleen at Day 4 and Day 14 (Fig. S4B). Under this continuous depletion schedule, we first assessed the impact of cell depletion on the tumor inhibitory effect of osimertinib monotherapy *in vivo*. During the 14‐day osimertinib treatment period, no significant differences in tumor volume were observed among the control, anti‐NK1.1, and anti‐CD8 antibody groups. However, tumor regrowth was observed significantly earlier in the anti‐CD8 antibody‐treated mice compared to the anti‐NK1.1 or control group. In contrast, there was no difference in tumor regrowth in the anti‐NK1.1 antibody group compared with the control group (Fig. 3A), suggesting a significant contribution of CD8^+^ cells, but a limited role for NK1.1^+^ cells, in the osimertinib‐induced antitumor effect.

Next, we assessed whether NK1.1^+^ or CD8^+^ cells contribute to the antitumor effect in the tumor treated with the combination of osimertinib and ADU‐S100 *in vivo*. The trends observed during osimertinib treatment were similar to those described previously (Fig. 2C). However, tumor regrowth was observed significantly earlier in both the anti‐NK1.1 antibody and anti‐CD8 antibody groups compared with the control group (Fig. 3B). These results suggest that both NK1.1^+^ and CD8^+^ cells contribute to the enhanced antitumor effect observed with the combination therapy of osimertinib and ADU‐S100. Consistently, an increase in the expression of Granzyme B was observed in the stromal area of tumors from mice treated with the combination therapy at Day 4 compared to those treated with osimertinib monotherapy (Fig. 3C).

To investigate whether ADU‐S100 targets cancer cells or stromal cells *in vivo*, we assessed the expression of IRF3, a protein downstream of the STING pathway, on serial sections of tumors from the mice treated with osimertinib and/or ADU‐S100 harvested on Day 4. Minimal IRF3 staining was observed in stromal cells of tumors treated with osimertinib monotherapy. In contrast, pronounced total IRF3 staining was observed in EGFR‐positive areas—corresponding *Egfr*‐mutant lung cancer cells—in mice receiving combination therapy (Fig. 3D). To verify that this increased expression reflects functional STING pathway activation rather than protein upregulation alone, we evaluated the phosphorylation of IRF3. In parallel with the increase in total IRF3, the p‐IRF3‐positive area was significantly increased in the combination group, indicating activation of the STING–IRF3 axis within the tumor tissue (Fig. S4C).

Finally, to evaluate the direct inhibitory effect of ADU‐S100 on cancer cells, we performed an *in vitro* viability assay. Human cell lines (PC‐9 and H1975) were treated with DMSO, osimertinib, ADU‐S100, or their combination. ADU‐S100 alone did not significantly inhibit cell proliferation compared to the DMSO control, and no significant additional growth‐inhibitory effect was observed when combined with osimertinib (Fig. 3E). To assess whether ADU‐S100 engages the STING pathway in these cancer cells despite the lack of growth inhibition, we stained the *in vitro* cell blocks for IRF3. IRF3 expression was significantly higher in cells treated with ADU‐S100 compared to those treated with the control (Fig. S4D). To assess whether similar results were observed in a murine cell line, we further evaluated the murine *Egfr*‐mutant cell line (mDEL) derived from our syngeneic tumor model [15]. Consistent with findings in human cell lines, ADU‐S100 monotherapy did not induce growth inhibition in mDEL cells, and combination with osimertinib yielded no additional growth‐inhibitory effects (Fig. S4E). Taken together, these results suggest that the STING agonist exerts its antitumor effect primarily through indirect mechanisms, such as tumor immunity involving CD8^+^ and NK cells, rather than through direct cytotoxicity to tumor cells.

### 3.4 Local administration of ADU‐S100 in combination with osimertinib‐induced abscopal effect in *Egfr*‐mutant tumors

**Fig. 4:**
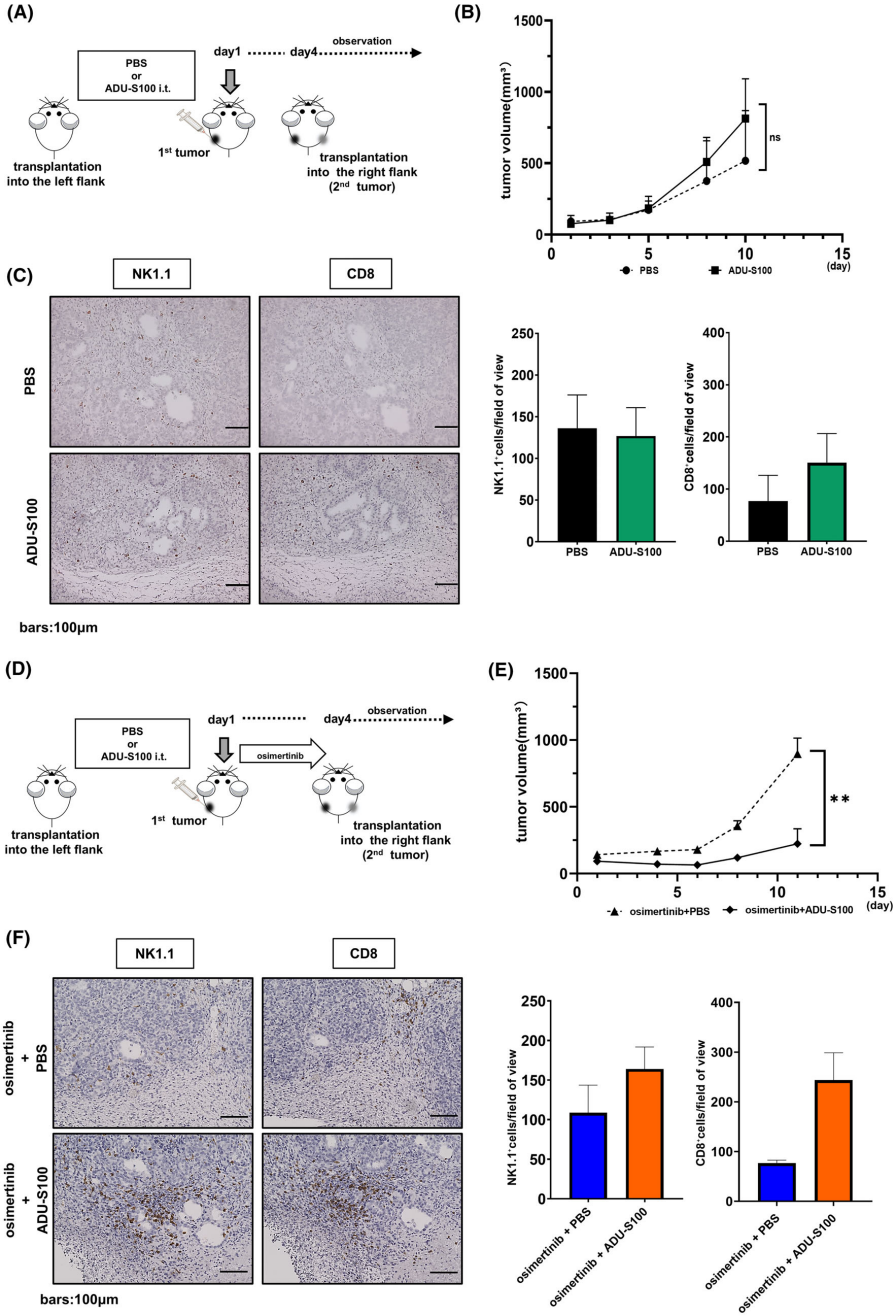
Abscopal effects induced by osimertinib and local injection of ADU‐S100 in *Egfr*‐mutant lung cancer mouse model. (A) Schematic image of the transplantation and treatment schedule of the *Egfr*‐mutant lung cancer mouse model. 1st = first, 2nd = secondary. (B) Secondary tumor growth *n* = 3 tumors per group, 3 mice per group. Data shown are representative of two independent experiments with similar results. Error bars represent the standard error. ns = not statistically significant, Student's *t*‐test. (C) Representative images of NK1.1 and CD8 immunohistochemistry (IHC) staining on secondary tumors, 7 days after transplantation. The NK1.1^+^ and CD8^+^ cells were quantified using imagej software. Error bars represent the standard error (*n* = 5 fields of view per group). Scale bars: 100 μm. Data shown are representative of two independent experiments with similar results. Serial sections of the same tumor tissue were used for the various immunostainings shown in this panel. (D) Schematic image of the transplantation and treatment schedule of the *Egfr*‐mutant lung cancer mouse model with osimertinib combination. 1st = first, 2nd = secondary. (E) Secondary tumor growth *n* = 4 tumors per group, 4 mice per group. Data shown are representative of three independent experiments with similar results. Error bars represent the standard error. **P < 0.01, Student's *t*‐test. (F) Representative images of NK1.1 and CD8 IHC staining on the secondary tumors, 7 days after transplantation. The NK1.1^+^ and CD8^+^ cells were quantified using the imagej software. Error bars represent the standard error (*n* = 7 fields of view per group). Scale bars: 100 μm. Data shown are representative of two independent experiments with similar results. Serial sections of the same tumor tissue were used for the various immunostainings shown in this panel.

Given that previous studies have shown that ADU‐S100 induces systemic immunity with an abscopal effect [27, 28], we investigated whether the combination therapy of osimertinib and ADU‐S100 could induce an abscopal effect in an *Egfr*‐mutant lung cancer mouse model with a noninflamed TME. To assess the abscopal effect of ADU‐S100 monotherapy, we utilized a bilateral tumor model [27]. In this model, tumor cells were transplanted into the left flank (1st tumor), and subsequently, mice received intratumoral injections of PBS or ADU‐S100. Three days after the injection, a second tumor was transplanted on the contralateral flank (2nd tumor) and tumor growth was monitored (Fig. 4A). The growth of the 2nd tumor following the injection of ADU‐S100 monotherapy to 1st tumors was not significantly different from that following PBS (Fig. 4B). NK1.1^+^ and CD8^+^ cells infiltration remained comparable between the treatment groups (Fig. 4C). These results suggest that ADU‐S100 monotherapy is insufficient to induce abscopal effects in the *Egfr*‐mutant lung cancer mouse model. We also assessed the role of osimertinib in the abscopal effect for the 2nd tumor; however, no superior inhibitory effect for the 2nd tumors was observed in the mice bearing 1st tumors treated with osimertinib compared with those treated with PBS (Fig. S5A,B). No significant difference in the infiltration of NK1.1^+^ and CD8^+^ cells was observed in the TME of the 2nd tumors between the mice treated with osimertinib or PBS (Fig. S5C), suggesting that osimertinib monotherapy does not induce an abscopal effect.

We then assessed the role of combination therapy with osimertinib and ADU‐S100 in inducing an abscopal effect. In this setting, the 1st tumor was treated intratumorally with either PBS or ADU‐S100 on Day 1, with osimertinib administered for 3 days starting on the day of intratumoral administration. Three days after the intratumoral administration, the 2nd tumor was transplanted (Fig. 4D). Notably, a significant difference was observed in the growth of the 2nd tumor with the combination of osimertinib and ADU‐S100 compared with that treated with osimertinib and PBS (Fig. 4E).

**Fig. 5:**
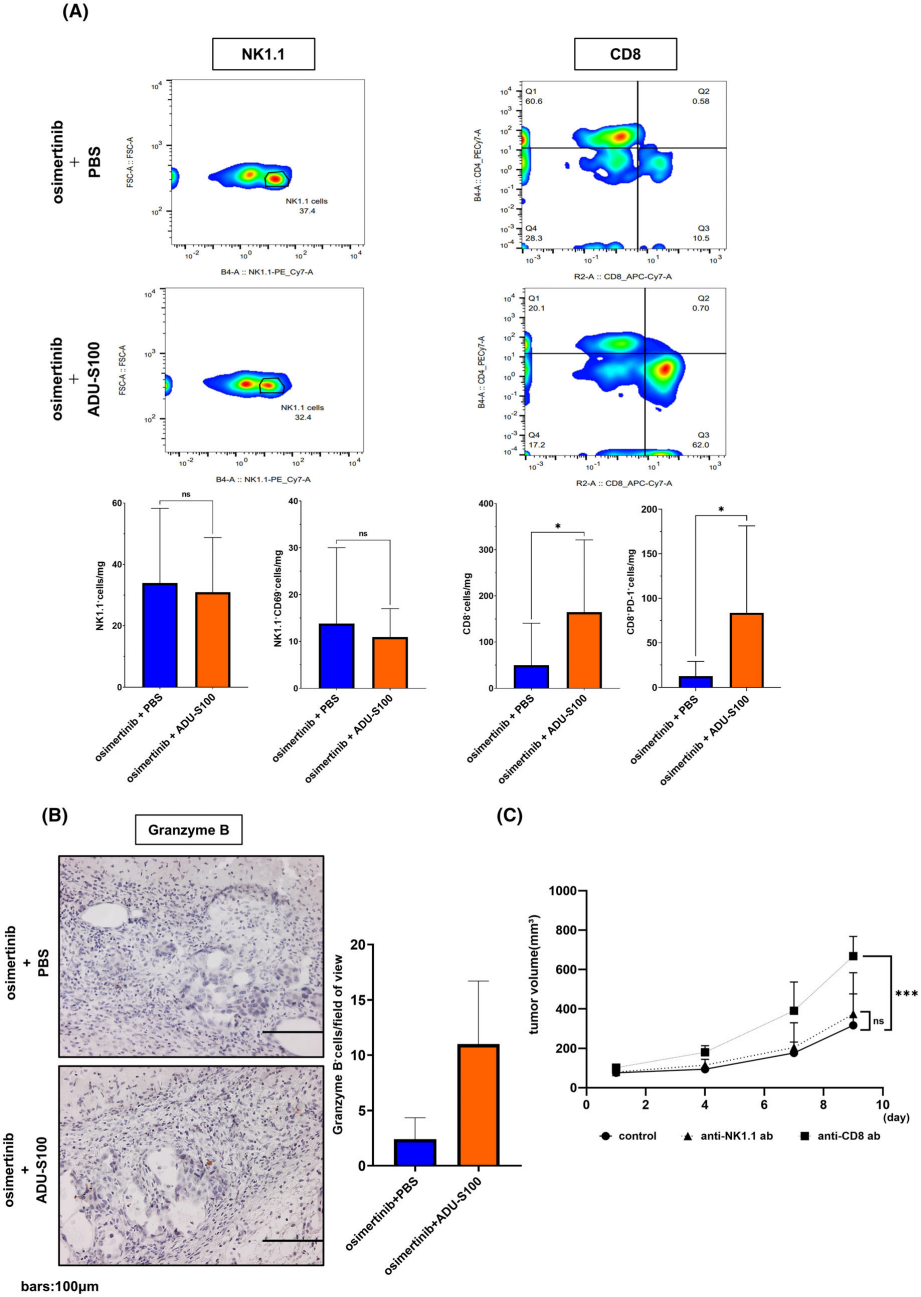
Abscopal effect induced by osimertinib and ADU‐S100 combination therapy is mediated by CD8^+^ cells. (A) Flow cytometry analysis of NK1.1^+^ and CD8^+^ cells isolated from secondary tumors, 7 days after transplantation. For NK1.1^+^ cells, *n* = 8 per group; for CD8^+^ cells, *n* = 12 per group. Error bars represent the standard error. ns = not significant, *P < 0.05, Student's *t*‐test. The NK1.1^+^ cell data are the integrated results of two independent experiments with matching conditions. The CD8^+^ cell data are the integrated results of three independent experiments with matching conditions. (B) Representative images of Granzyme B immunohistochemistry staining on the secondary tumors, 7 days after transplantation. The Granzyme B^+^ cells were quantified using the imagej software. Error bars represent the standard error (*n* = 5 fields of view per group). Scale bars: 100 μm. Data shown are representative of two independent experiments with similar results. (C) Secondary tumor growth with anti‐CD8 or anti‐NK1.1 antibodies. For NK1.1^+^ cells and CD8^+^ cells, *n* = 8 tumors from 8 mice per group; for Control, *n* = 6 tumors from six mice per group. The data are the integrated results of two independent experiments with matching conditions. Error bars represent the standard error. ns = not significant, ***P < 0.001, Student's *t*‐test.

To elucidate the mechanisms driving this systemic response, we evaluated immunogenic cell death (ICD) markers in the 1st tumor on Day 4. IHC analysis revealed a nuclear‐to‐cytoplasmic translocation of HMGB1, indicative of its release, and an increase in CRT expression in the ADU‐S100 monotherapy and combination groups compared to the vehicle and osimertinib monotherapy groups (Fig. S5D), suggesting that local ADU‐S100 administration induced ICD markers within the primary tumor. However, given that ADU‐S100 monotherapy alone was insufficient to control distant tumors (Fig. 4B), we next evaluated the TME of the 2nd tumor to determine how the combination therapy uniquely facilitates systemic tumor control. IHC analysis revealed NK1.1^+^ cell infiltration was similar between the two treatment groups. In contrast, an increase in CD8^+^ cell infiltration was observed in the 2nd tumors of mice treated with the combination therapy compared to those treated with osimertinib and PBS (Fig. 4F). FCM analysis revealed no significant differences in CD69 expression among NK1.1^+^ cells between the two treatment groups; however, the proportion of PD‐1^+^ CD8^+^ T cells was significantly increased in the combination group (Fig. 5A). Since PD‐1 expression can reflect both activation and exhaustion, we evaluated the expression of cytotoxic effector molecules within the 2nd tumor. IHC analysis showed an increase in Granzyme B^+^ cells within the 2nd tumor of the combination group (Fig. 5B). This cytotoxic marker, combined with the PD‐1 upregulation, suggests that the recruited CD8^+^ cells are functional effector cells with cytotoxic potential. To ascertain the functional role of NK1.1^+^ and CD8^+^ cells, we performed depletion experiments using antibodies against NK1.1^+^ or CD8^+^ cells. The anti‐NK1.1 antibody‐treated group did not show a significant difference in tumor growth compared to the control group. However, the group treated with anti‐CD8 antibody exhibited a significant reduction in the antitumor effect compared with the control group (Fig. 5C). These results demonstrate that the abscopal effect induced by the combination therapy is primarily mediated by activated CD8^+^ cells.

## 4 Discussion

Recent reports suggest the ability of EGFR‐TKIs to elicit antitumor immunity [29, 30, 31], prompting investigation into their potential in combination with ICIs. However, the risk of severe adverse events, such as interstitial lung disease (ILD), associated with EGFR‐TKI/ICI combinations limits their clinical application [32, 33, 34]. This study provides the first evidence that combining osimertinib with a focal intratumoral injection of STING agonist augments osimertinib‐induced immunity through the activation of CD8^+^ T cells and NK cells, leading to an abscopal effect.

Previous studies showed minimal STING pathway activation in *EGFR*‐mutant lung cancer cells treated with EGFR‐TKIs [35]. Consistent with these findings, *Egfr*‐mutant mouse tumors treated with osimertinib monotherapy showed minimal IRF3 expression. However, combining osimertinib with ADU‐S100 resulted in an increase in both total IRF3 expression and its phosphorylation (p‐IRF3), indicating activation of the STING pathway, which is an established driver for the enhancement of antitumor immunity [10]. Consistently, in the primary tumor, ADU‐S100 administration induced ICD markers, specifically increased CRT expression and nuclear‐to‐cytoplasmic translocation of HMGB1, indicating local induction of ICD‐associated features.

*EGFR*‐mutant lung cancer is characterized by low expression of cancer antigens and a non‐inflamed TME, which may hinder effective antigen presentation and T‐cell recognition [36]. In this study, we observed an increase in PD‐1^+^ CD8^+^ cells in the secondary tumor distant from the ADU‐S100 injection site of mice treated with combination therapy, unlike monotherapy using osimertinib or a STING agonist. While intratumorally injected drugs carry the potential for systemic leakage, the failure of ADU‐S100 monotherapy to suppress the secondary tumor and the absence of NK cell infiltration in the distant tumor, unlike that observed at the primary site, argue against local activation by leaked drugs. Furthermore, the abrogation of the abscopal effect upon CD8^+^ cell depletion indicates that secondary tumor regression is primarily an adaptive immune response dependent on tumor‐reactive CD8^+^ T cells. The increase in Granzyme B^+^ cells within the distant tumor further supports that these recruited CD8^+^ T cells are functional effectors with cytotoxic activity.

Notably, we observed that while local induction of ICD markers was achieved by ADU‐S100 alone, a systemic abscopal effect required the addition of osimertinib. We and others previously demonstrated that oncogenic EGFR signaling actively maintains an immunosuppressive TME, making EGFR inhibition a crucial factor for inducing CD8^+^ T‐cell responses [7, 8]. Consistent with this, our current findings suggest that while ADU‐S100‐induced ICD provides an important immunological trigger, the concurrent reversal of EGFR‐driven immunosuppression by osimertinib is likely required to translate this local trigger into a systemic CD8^+^ T‐cell response and the subsequent abscopal effect. Although NK cell infiltration was not increased in the secondary tumors, the local NK cell and dendritic cell infiltration induced by osimertinib and ADU‐S100 in the primary tumors may contribute to the overall immune activation observed with the combination therapy [25, 26].

Our findings demonstrate that osimertinib treatment increases MHC class I expression on tumor cells; however, the expression levels remain lower than those in normal spleen cells. This suggests that NK cells may retain the potential to recognize these tumor cells based on the missing‐self hypothesis [24]. Although osimertinib alone did not significantly enhance NK cell infiltration into the tumor, this observation provides a rationale for our hypothesis that stimulating NK cell activity through a STING agonist could augment the antitumor efficacy of osimertinib. Therefore, the combination therapy effectively addresses two key limitations of osimertinib monotherapy: the insufficient recruitment of NK cells and the lack of robust activation of the innate immune response.

Our findings could provide a novel treatment strategy not only in patients with advanced stage but also in those with early‐stage lung cancer. Perioperative immunotherapy for lung cancer has rapidly advanced [37, 38]; however, its efficacy in *EGFR*‐mutant lung cancer remains unclear. Currently, postoperative administration of osimertinib has been approved for stage II–III lung cancer harboring an *EGFR* mutation [39]. Based on our findings, the combination therapy of osimertinib and a STING agonist may offer a promising new therapeutic approach for the perioperative management of *EGFR*‐mutant lung cancer.

This study has several limitations. First, it was conducted in a mouse model and focused on a specific type of *Egfr*‐mutant lung cancer. Second, our evaluation of the TME was limited to specific immune cell populations, such as CD8^+^ T cells and NK cells. Furthermore, the combination of osimertinib and ADU‐S100 did not result in complete tumor eradication in our mouse model, suggesting potential limitations in immune enhancement. Third, the STING agonist was administered as a single intratumoral injection. A preclinical study reported that repeated administrations of a STING agonist suppressed distant tumors long term by inducing memory‐like NK cells *in vivo* [40], suggesting that repeated dosing of ADU‐S100 in our mouse model might have resulted in a stronger antitumor effect. However, repeated intratumoral injections would not be feasible in a clinical setting. Fourth, we employed an osimertinib cessation protocol to evaluate the immune‐mediated response, which would otherwise be masked by its potent direct tumor‐suppressive effects. Although continuous osimertinib administration is the clinical standard, the immune activation induced by the combination treatment during the active treatment period suggests that adding a STING agonist may further augment the overall therapeutic efficacy of continuous osimertinib treatment.

## 5 Conclusions

This study demonstrates that ADU‐S100 augments osimertinib‐induced antitumor immunity by activating both innate and adaptive immune responses. The observed abscopal effect highlights the potential of this combination therapy as a novel immunotherapy for *EGFR*‐mutant lung cancer.

## Conflict of interest

Johannes Brägelmann has received research fund from Bayer. outside of the submitted work. Kiichiro Ninomiya has received honoraria from AstraZeneca K.K., Nippon Boehringer Ingelheim Co., Ltd., Kyowa Kirin Co., Ltd., Eli Lilly Japan K.K., Chugai Pharmaceutical Co., Ltd., Nippon Kayaku Co., Ltd., TAIHO PHARMACEUTICAL CO., LTD., MSD K.K., Ono Pharmaceutical CO., LTD., Takeda Pharmaceutical CO., LTD., Pfizer Japan Inc., Bristol Myers Squibb K.K., Elekta K.K., Janssen Pharmaceutical K.K., Amgen K.K., Novartis Pharma K.K., Guardant Health Japan Corp., CareNet Inc. and Daiichi Sankyo Co., Ltd. outside the submitted work. Katsuyuki Hotta has received honoraria from AstraZeneca K.K., Chugai Pharmaceutical Co., Ltd., Eli Lilly Japan K.K., MSD K.K., Bristol Myers Squibb K.K., Ono Pharmaceutical Co., Ltd., Nippon Boehringer‐Ingelheim Co., Ltd., Nippon Kayaku Co., Ltd., Amgen K.K., TAIHO PHARMACEUTICAL CO., LTD., Merck Biopharma Co., Ltd.; and research fund from MSD K.K., AstraZeneca K.K., Chugai Pharmaceutical Co., Ltd., Eli Lilly Japan K.K., Bristol Myers Squibb K.K., Ono Pharmaceutical Co., Ltd., and Abbvie Inc. outside the submitted work. Yosuke Togashi has received honoraria from Ono Pharmaceutical Co. Ltd., Bristol Myers Squibb K.K., Chugai Pharmaceutical Co., AstraZeneca K.K., Eisai Co. Ltd. and MSD K.K.; and research fund from Daiichi Sankyo Co., Ltd., Janssen Pharmaceutical K.K., AstraZeneca K.K., KORTUC Inc., Takeda Pharmaceutical CO., LTD. and TAIHO PHARMACEUTICAL CO., LTD. outside the submitted work. Martin L. Sos has received research fund from PearlRiver Bio. Outside the submitted work. Kadoaki Ohashi has received honoraria from Eli Lilly Japan K.K., Novartis Pharma K.K., Chugai Pharmaceutical Co., Ltd., AstraZeneca K.K., Kyowa Kirin Co., Ltd., and Novartis Pharma K.K; grants from Nippon Boehringer Ingelheim Co., Ltd., Chugai Pharmaceutical Co., Ltd., Eli Lilly Japan K.K., Daiichi Sankyo Co., Ltd., Amgen K.K., and Novartis Pharma K.K; and receipt of research reagents from Genentech, Inc. and Novartis Pharma K.K. outside the submitted work. Jun Nishimura, Tadahiro Kuribayashi, Sachi Okawa, Masataka Taoka, Shunta Mori, Tomoka Nishimura, Takaaki Tanaka, Go Makimoto, Kammei Rai, Eiki Ichihara, Ryohei Katayama, Masahiro Tabata, Yoshinobu Maeda, Katusyuki Kiura declare no potential conflict of interest.

## Author contributions

J.N. contributed to acquisition of data, analysis and interpretation of data, writing—original draft, writing—review and editing. T.K. contributed to acquisition of data, analysis and interpretation of data, writing—review and editing. J.B. contributed to acquisition of data, analysis and interpretation of data, writing—review and editing. S.O. contributed to acquisition of data, writing—review and editing. M. Taoka contributed to acquisition of data, writing—review and editing. S.M. contributed to acquisition of data, writing—review and editing. T.N. contributed to acquisition of data, writing—review and editing. T.T. contributed to acquisition of data, writing—review and editing. G.M. contributed to resources, writing—review and editing. K.N. contributed to resources, writing—review and editing. K.R. contributed to resources, writing—review and editing. E.I. contributed to resources, writing—review and editing. R.K. contributed to acquisition of data, resources, writing—review and editing. K.H. contributed to resources, writing—review and editing. M. Tabata contributed to resources, writing—review and editing. Y.T. contributed to resources, writing—review and editing. Y.M. contributed to resources, writing—review and editing. M.L.S. contributed to analysis and interpretation of data, writing—review and editing. K.K. contributed to analysis and interpretation of data, writing—review and editing. K.O. contributed to conception and design of the study, analysis and interpretation of data, writing—original draft, writing—review and editing.

## Supporting information


**Fig. S1.** Immune profiling of the tumor microenvironment in *Egfr*‐mutant lung tumors treated with EGFR inhibitors. (A) Gene Set Enrichment Analysis was performed using RNA‐sequencing data (Fig. 1B) in untreated mouse tumors compared to those treated with gefitinib or osimertinib for 14 days. The Hallmark IFN‐γ response signature was most upregulated in the treated group, with a normalized enrichment score of 9.96 (adj. *P* < 0.0001). NES = normalized enrichment score. (B) Validation of immune‐related gene expression by RT‐qPCR. Relative mRNA expression of *Cd8a*, *Ifng*, *Gzmb*, and *Cxcl9* in tumor tissues treated with osimertinib for 0, 4, and 14 days (*n* = 4 per group). Data were normalized to *Gapdh*. Error bars represent the standard error. ns = not significant, *P < 0.05; **P < 0.01, ***P < 0.001, one‐way analysis of variance (ANOVA) with *post hoc* Tukey test.

**Fig. S2.** Functional relevance of EGFR‐TKI‐induced immune modulation. (A) Representative flow cytometry data showing the expression of MHC class I proteins H‐2Kb and H‐2Db within the spleen and dissociated tumor cells. (B) robust coefficient of variation (rCV) (Day 0: *n* = 12 per group, Day 4: *n* = 10 per group) for H‐2Db and H‐2Kb are shown. Error bars represent the standard error. ns = not significant, Student's *t*‐test. (C) Representative images of Granzyme B immunohistochemistry (IHC) staining on tumors from *Egfr*‐mutant mice treated with osimertinib (15 mg·kg^−1^·day^−1^, via oral gavage [p.o.]) after 0 or 4 days. The Granzyme B⁺ cells were quantified using imagej software. Error bars represent the standard error (*n* = 5 fields of view per group). Scale bars: 100 μm. ***P < 0.001, Student's *t*‐test.

**Fig. S3.** Immune cell infiltration in *Egfr*‐mutant lung tumors following osimertinib and ADU‐S100 treatment. Representative immunohistochemistry (IHC) staining images of NK1.1, CD8, and CD11c on *Egfr*‐mutant lung tumors from mice treated with osimertinib (15 mg·kg^−1^·day^−1^, via oral gavage [p.o.], 7 days/week) and combination of osimertinib (15 mg·kg^−1^·day^−1^, p.o., 7 days/week) and ADU‐S100 (50 μg, intratumorally, Day 1) for 14 days and after 3 days of drug withdrawal. The NK1.1⁺, CD8⁺, and CD11c⁺ cells were quantified using imagej software. Error bars represent the standard error (*n* = 5 fields of view per group). Scale bars: 100 μm. *P < 0.05; **P < 0.01, Student's *t*‐test.

**Fig. S4.** Efficiency of immune cell depletion and evaluation of the direct effects of ADU‐S100 *in vitro*. (A) Schematic image of the experimental schedule for immune cell depletion. (B) Representative immunohistochemistry (IHC) staining image of CD8 and NK1.1 in spleen tissues harvested on Day 4, Day 14 and untreated control. Scale bars: 100 μm. (C) Representative images of p‐IRF3 IHC staining on *Egfr*‐mutant lung cancer tumors from mice treated with osimertinib (15 mg·kg^−1^·day^−1^, via oral gavage [p.o.]) and combination of osimertinib (15 mg·kg^−1^·day^−1^, p.o.) and ADU‐S100 (50 μg, intratumoral administration, Day 1) for 4 days. %Area of p‐IRF3⁺ cells was quantified using imagej software. Error bars represent the standard error (*n* = 5 fields of view per group). Scale bars: 100 μm. ***P < 0.001, Student's *t*‐test. (D) Representative images of IRF3 IHC staining after 4 days of DMSO or ADU‐S100 (10 μmol·L^−1^) load. IRF3⁺ areas (%) were quantified using imagej software. Error bars represent the standard error (*n* = 5 fields of view per group). Scale bars: 100 μm. *P < 0.05, **P < 0.01, Student's *t*‐test. (E) Crystal violet assay of the murine *Egfr*‐mutant cell line (mDEL). Cancer cells were treated with 0.2% dimethyl sulfoxide, 0.1 μmol·L^−1^ osimertinib and/or 10 μmol·L^−1^ ADU‐S100 for 4 days.

**Fig. S5.** Absence of abscopal effect with osimertinib monotherapy and ADU‐S100‐induced immunogenic cell death in *Egfr*‐mutant lung tumors. (A) Schematic image of the treatment and transplantation schedule. (B) Secondary tumor growth. *n* = 4 tumors per group, 4 mice per group. Data shown are representative of two independent experiments with similar results. Error bars represent the standard error. ns = not significant, Student's *t*‐test. (C) Representative images of NK1.1 and CD8 immunohistochemistry (IHC) staining on secondary tumors 7 days after transplantation. The NK1.1⁺ and CD8⁺ cells were quantified using imagej software. Error bars represent the standard error (*n* = 5 fields of view per group). Scale bars: 100 μm. ns = not significant, Student's *t*‐test. (D) Representative images of HMGB1 and calreticulin (CRT) IHC staining on first tumors from mice treated with PBS (100 μL, intratumoral administration [i.t.], Day 1), osimertinib (15 mg·kg^−1^·day^−1^, oral gavage [p.o.]), ADU‐S100 (50 μg, i.t., Day 1), and combination of osimertinib (15 mg·kg^−1^·day^−1^, p.o.) and ADU‐S100 (50 μg, i.t., Day 1) for 4 days. Scale bars: 100 μm.

## Data Availability

The datasets used and/or analyzed during the current study are available from the corresponding author upon reasonable request.

## References

[1] Saito M , Shiraishi K , Kunitoh H , Takenoshita S , Yokota J , Kohno T . Gene aberrations for precision medicine against lung adenocarcinoma. Cancer Sci. 2016;107(6):713–720. 10.1111/cas.12941 27027665 PMC4968599

[2] Ohashi K , Maruvka YE , Michor F , Pao W . Epidermal growth factor receptor tyrosine kinase inhibitor–resistant disease. J Clin Oncol. 2013;31(8):1070–1080. 10.1200/JCO.2012.43.3912 23401451 PMC3589701

[3] Passaro A , Jänne PA , Mok T , Peters S . Overcoming therapy resistance in EGFR‐mutant lung cancer. Nat Can. 2021;2(4):377–391. 10.1038/s43018-021-00195-8 35122001

[4] Ohashi K , Ninomiya K , Yoshioka H , Bessho A , Shibayama T , Aoe K , et al. Impact of HER2 expression on EGFR‐TKI treatment outcomes in lung tumors harboring EGFR mutations: a HER2‐CS study subset analysis. Lung Cancer. 2020;150:83–89. 10.1016/j.lungcan.2020.09.024 33096420

[5] Reck M , Remon J , Hellmann MD . First‐line immunotherapy for non–small‐cell lung cancer. J Clin Oncol. 2022;40(6):586–597. 10.1200/JCO.21.01497 34985920

[6] Lee CK , Man J , Lord S , Links M , Gebski V , Mok T , et al. Checkpoint inhibitors in metastatic EGFR‐mutated non–small cell lung cancer – a meta‐analysis. J Thorac Oncol. 2017;12(2):403–407. 10.1016/j.jtho.2016.10.007 27765535

[7] Sugiyama E , Togashi Y , Takeuchi Y , Shinya S , Tada Y , Kataoka K , et al. Blockade of EGFR improves responsiveness to PD‐1 blockade in EGFR‐mutated non‐small cell lung cancer. Sci Immunol. 2020;5(43):eaav3937. 10.1126/sciimmunol.aav3937 32005679

[8] Nishii K , Ohashi K , Tomida S , Nakasuka T , Hirabae A , Okawa S , et al. CD8+ T‐cell responses are boosted by dual PD‐1/VEGFR2 blockade after EGFR inhibition in Egfr‐mutant lung cancer. Cancer Immunol Res. 2022;10(9):1111–1126. 10.1158/2326-6066.CIR-21-0751 35802887

[9] Qiao M , Jiang T , Liu X , Mao S , Zhou F , Li X , et al. Immune checkpoint inhibitors in EGFR‐mutated NSCLC: dusk or dawn? J Thorac Oncol. 2021;16(8):1267–1288. 10.1016/j.jtho.2021.04.003 33915248

[10] Corrales L , McWhirter SM , Dubensky TW , Gajewski TF . The host STING pathway at the interface of cancer and immunity. J Clin Invest. 2016;126(7):2404–2411. 10.1172/JCI86892 27367184 PMC4922692

[11] Zhang Z , Zhang C . Regulation of cGAS–STING signalling and its diversity of cellular outcomes. Nat Rev Immunol. 2025;25:1–20. 10.1038/s41577-024-01112-7 39774812

[12] Jiang M , Chen P , Wang L , Li W , Chen B , Liu Y , et al. cGAS‐STING, an important pathway in cancer immunotherapy. J Hematol OncolJ Hematol Oncol. 2020;13(1):81. 10.1186/s13045-020-00916-z 32571374 PMC7310007

[13] Nicolai CJ , Wolf N , Chang IC , Kirn G , Marcus A , Ndubaku CO , et al. NK cells mediate clearance of CD8+ T cell‐resistant tumors in response to STING agonists. Sci Immunol. 2020;5(45):eaaz2738. 10.1126/sciimmunol.aaz2738 32198222 PMC7228660

[14] Gehrcken L , Deben C , Smits E , Van Audenaerde JRM . STING agonists and how to reach their full potential in cancer immunotherapy. Adv Sci. 2025;12(17):2500296. 10.1002/advs.202500296 PMC12061341 40145387

[15] Fujitani M , Shimomura, I , Takemoto, A , Ohashi K , Katayama R . Exploration of EGFR‐TKI resistance mechanisms using a syngeneic mouse model of EGFR‐mutant lung cancer. Program Abstr 47th Annu Meet Mol Biol Soc Jpn Web. 2024;2P–880P.

[16] Higo H , Ohashi K , Makimoto G , Nishii K , Kudo K , Kayatani H , et al. EGFR‐TKI acquired resistance in lung cancers harboring EGFR mutations in immunocompetent C57BL/6J mice. Lung Cancer. 2019;136:86–93. 10.1016/j.lungcan.2019.08.019 31470227

[17] Nakasuka T , Ohashi K , Nishii K , Hirabae A , Okawa S , Tomonobu N , et al. PD‐1 blockade augments CD8+ T cell dependent antitumor immunity triggered by ad‐SGE‐REIC in Egfr‐mutant lung cancer. Lung Cancer Amst Neth. 2023;178:1–10. 10.1016/j.lungcan.2023.01.018 36753780

[18] Kudo K , Ohashi K , Makimoto G , Higo H , Kato Y , Kayatani H , et al. Triplet therapy with afatinib, cetuximab, and bevacizumab induces deep remission in lung cancer cells harboring EGFR T790M in vivo. Mol Oncol. 2017;11(6):670–681. 10.1002/1878-0261.12063 28388009 PMC5467494

[19] Makimoto G , Ohashi K , Tomida S , Nishii K , Matsubara T , Kayatani H , et al. Rapid acquisition of alectinib resistance in ALK‐positive lung cancer with high tumor mutation burden. J Thorac Oncol. 2019;14(11):2009–2018. 10.1016/j.jtho.2019.07.017 31374369

[20] Müller N , Lorenz C , Ostendorp J , Heisel FS , Friese UP , Cartolano M , et al. Characterizing evolutionary dynamics reveals strategies to exhaust the spectrum of subclonal resistance in EGFR‐mutant lung cancer. Cancer Res. 2023;83(15):2471–2479. 10.1158/0008-5472.CAN-22-2605 37289018

[21] Brägelmann J , Lorenz C , Borchmann S , Nishii K , Wegner J , Meder L , et al. MAPK‐pathway inhibition mediates inflammatory reprogramming and sensitizes tumors to targeted activation of innate immunity sensor RIG‐I. Nat Commun. 2021;12(1):5505. 10.1038/s41467-021-25728-8 34535668 PMC8448826

[22] Petitprez F , Levy S , Sun CM , Meylan M , Linhard C , Becht E , et al. The murine microenvironment cell population counter method to estimate abundance of tissue‐infiltrating immune and stromal cell populations in murine samples using gene expression. Genome Med. 2020;12(1):86. 10.1186/s13073-020-00783-w 33023656 PMC7541325

[23] Okawa S , Tomida S , Kuribayashi T , Nishimura J , Nakasuka T , Hirabae A , et al. Colony‐stimulating factor‐1 receptor inhibitor augments Osimertinib‐induced antitumor immunity via suppression of macrophages in lung cancer harboring EGFR mutation. Mol Cancer Ther. 2025;24(11):1763–1774. 10.1158/1535-7163.MCT-25-0002 40439218

[24] Guillerey C , Huntington ND , Smyth MJ . Targeting natural killer cells in cancer immunotherapy. Nat Immunol. 2016;17(9):1025–1036. 10.1038/ni.3518 27540992

[25] Li G , Zhao X , Zheng Z , Zhang H , Wu Y , Shen Y , et al. cGAS‐STING pathway mediates activation of dendritic cell sensing of immunogenic tumors. Cell Mol Life Sci CMLS. 2024;81(1):149. 10.1007/s00018-024-05191-6 38512518 PMC10957617

[26] Barry KC , Hsu J , Broz ML , Cueto FJ , Binnewies M , Combes AJ , et al. A natural killer‐dendritic cell axis defines checkpoint therapy‐responsive tumor microenvironments. Nat Med. 2018;24(8):1178–1191. 10.1038/s41591-018-0085-8 29942093 PMC6475503

[27] Sivick KE , Desbien AL , Glickman LH , Reiner GL , Corrales L , Surh NH , et al. Magnitude of therapeutic STING activation determines CD8+ T cell‐mediated anti‐tumor immunity. Cell Rep. 2018;25(11):3074–3085. 10.1016/j.celrep.2018.11.047 30540940

[28] Yang H , Lee WS , Kong SJ , Kim CG , Kim JH , Chang SK , et al. STING activation reprograms tumor vasculatures and synergizes with VEGFR2 blockade. J Clin Invest. 2019;129(10):4350–4364. 10.1172/JCI125413 31343989 PMC6763266

[29] Gurule NJ , McCoach CE , Hinz TK , Merrick DT , van Bokhoven A , Kim J , et al. A tyrosine kinase inhibitor‐induced interferon response positively associates with clinical response in EGFR‐mutant lung cancer. NPJ Precis Oncol. 2021;5(1):41. 10.1038/s41698-021-00181-4 34001994 PMC8129124

[30] Fang Y , Wang Y , Zeng D , Zhi S , Shu T , Huang N , et al. Comprehensive analyses reveal TKI‐induced remodeling of the tumor immune microenvironment in EGFR/ALK‐positive non‐small‐cell lung cancer. Onco Targets Ther. 2021;10(1):1951019. 10.1080/2162402X.2021.1951019 PMC8288040 34345533

[31] Lin Z , Wang Q , Jiang T , Wang W , Zhao JJ . Targeting tumor‐associated macrophages with STING agonism improves the antitumor efficacy of osimertinib in a mouse model of EGFR‐mutant lung cancer. Front Immunol. 2023;14:1077203. 10.3389/fimmu.2023.1077203 36817465 PMC9933873

[32] Creelan BC , Yeh TC , Kim SW , Nogami N , Kim DW , Chow LQM , et al. A phase 1 study of gefitinib combined with durvalumab in EGFR TKI‐naive patients with EGFR mutation‐positive locally advanced/metastatic non‐small‐cell lung cancer. Br J Cancer. 2021;124(2):383–390. 10.1038/s41416-020-01099-7 33012782 PMC7852511

[33] Riudavets M , Naigeon M , Texier M , Dorta M , Barlesi F , Mazieres J , et al. Gefitinib plus tremelimumab combination in refractory non‐small cell lung cancer patients harbouring EGFR mutations: the GEFTREM phase I trial. Lung Cancer. 2022;166:255–264. 10.1016/j.lungcan.2021.11.018 34953624

[34] Oxnard GR , Yang JCH , Yu H , Kim SW , Saka H , Horn L , et al. TATTON: a multi‐arm, phase Ib trial of osimertinib combined with selumetinib, savolitinib, or durvalumab in EGFR‐mutant lung cancer. Ann Oncol. 2020;31(4):507–516. 10.1016/j.annonc.2020.01.013 32139298

[35] Gong K , Guo G , Panchani N , Bender ME , Gerber DE , Minna JD , et al. EGFR inhibition triggers an adaptive response by co‐opting antiviral signaling pathways in lung cancer. Nat Can. 2020;1(4):394–409. 10.1038/s43018-020-0048-0 PMC7706867 33269343

[36] Chen DS , Mellman I . Oncology meets immunology: the cancer‐immunity cycle. Immunity. 2013;39(1):1–10. 10.1016/j.immuni.2013.07.012 23890059

[37] Felip E , Altorki N , Zhou C , Csőszi T , Vynnychenko I , Goloborodko O , et al. Adjuvant atezolizumab after adjuvant chemotherapy in resected stage IB‐IIIA non‐small‐cell lung cancer (IMpower010): a randomised, multicentre, open‐label, phase 3 trial. Lancet Lond Engl. 2021;398(10308):1344–1357. 10.1016/S0140-6736(21)02098-5 34555333

[38] Forde PM , Spicer J , Lu S , Provencio M , Mitsudomi T , Awad MM , et al. Neoadjuvant Nivolumab plus chemotherapy in resectable lung cancer. N Engl J Med. 2022;386(21):1973–1985. 10.1056/NEJMoa2202170 35403841 PMC9844511

[39] Wu Y‐L , Tsuboi M , He J , John T , Grohe C , Majem M , et al. Osimertinib in resected EGFR‐mutated non‐small‐cell lung cancer. N Engl J Med. 2020;383(18):1711–1723. 10.1056/NEJMoa2027071 32955177

[40] Khalifa AM , Nakamura T , Sato Y , Harashima H . Vaccination with a combination of STING agonist‐loaded lipid nanoparticles and CpG‐ODNs protects against lung metastasis via the induction of CD11bhighCD27low memory‐like NK cells. Exp Hematol Oncol. 2024;13(1):36. 10.1186/s40164-024-00502-w 38553761 PMC10981311

