# Supplementary figures and images for: Stimulator of interferon genes agonist augmented antitumor immunity of osimertinib in Egfr‐mutated lung cancer
Source: Mol Oncol. 2026 May 21:10.1002/1878-0261.70264. Online ahead of print. doi: 10.1002/1878-0261.70264 (PMC13398822; doi:10.1002/1878-0261.70264)

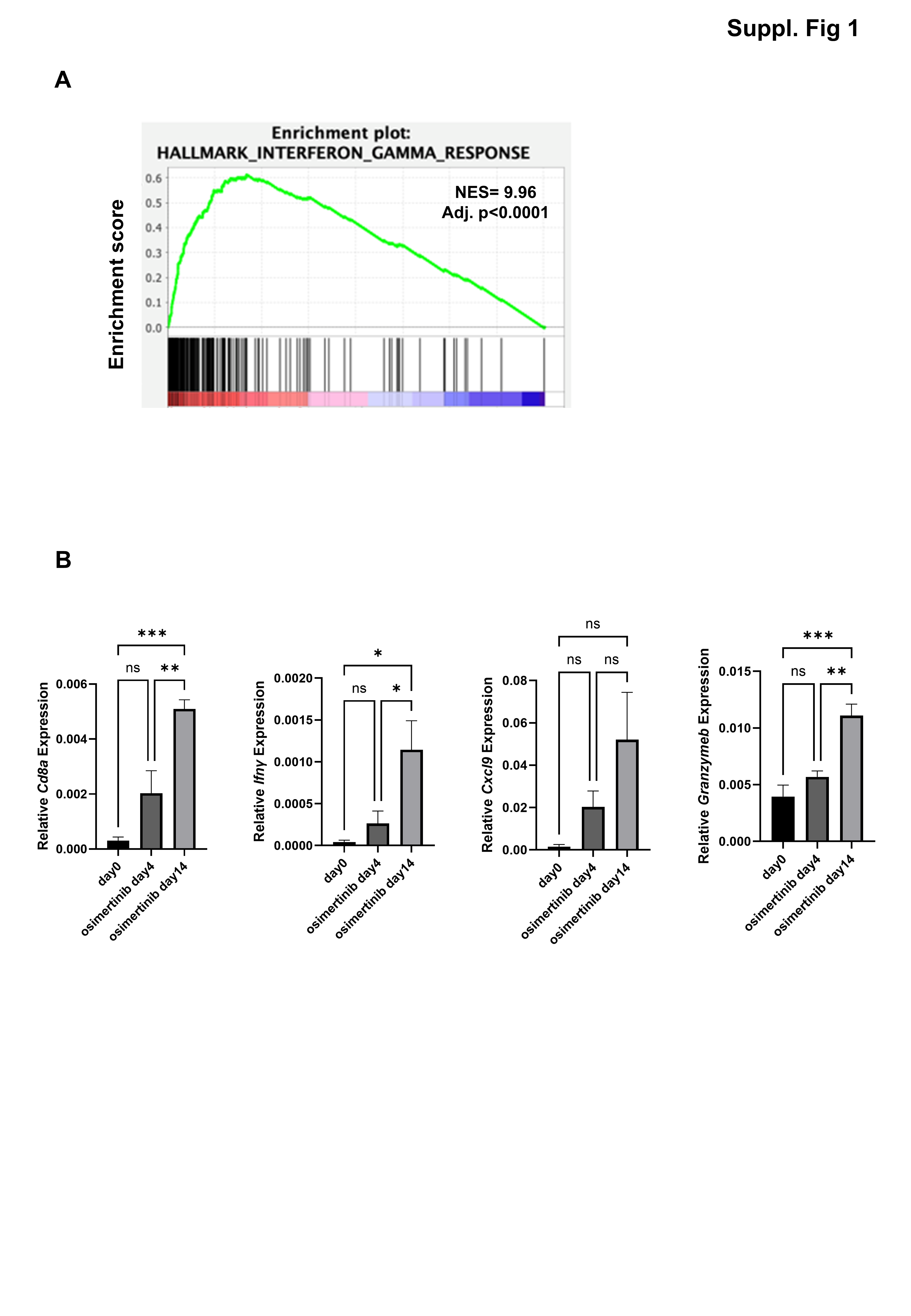

Supplement: Supplementary file 1 — Fig. S1. Immune profiling of the tumor microenvironment in Egfr‐mutant lung tumors treated with EGFR inhibitors. (A) Gene Set Enrichment Analysis was performed using RNA‐sequencing data (Fig. 1B) in untreated mouse tumors compared to those treated with gefitinib or osimertinib for 14 days. The Hallmark IFN‐γ response signature was most upregulated in the treated group, with a normalized enrichment score of 9.96 (adj. P < 0.0001). NES = normalized enrichment score. (B) Validation of immune‐related gene expression by RT‐qPCR. Relative mRNA expression of Cd8a, Ifng, Gzmb, and Cxcl9 in tumor tissues treated with osimertinib for 0, 4, and 14 days (n = 4 per group). Data were normalized to Gapdh. Error bars represent the standard error. ns = not significant, *P < 0.05; **P < 0.01, ***P < 0.001, one‐way analysis of variance (ANOVA) with post hoc Tukey test. [file MOL2-9999-0-s002.pdf]

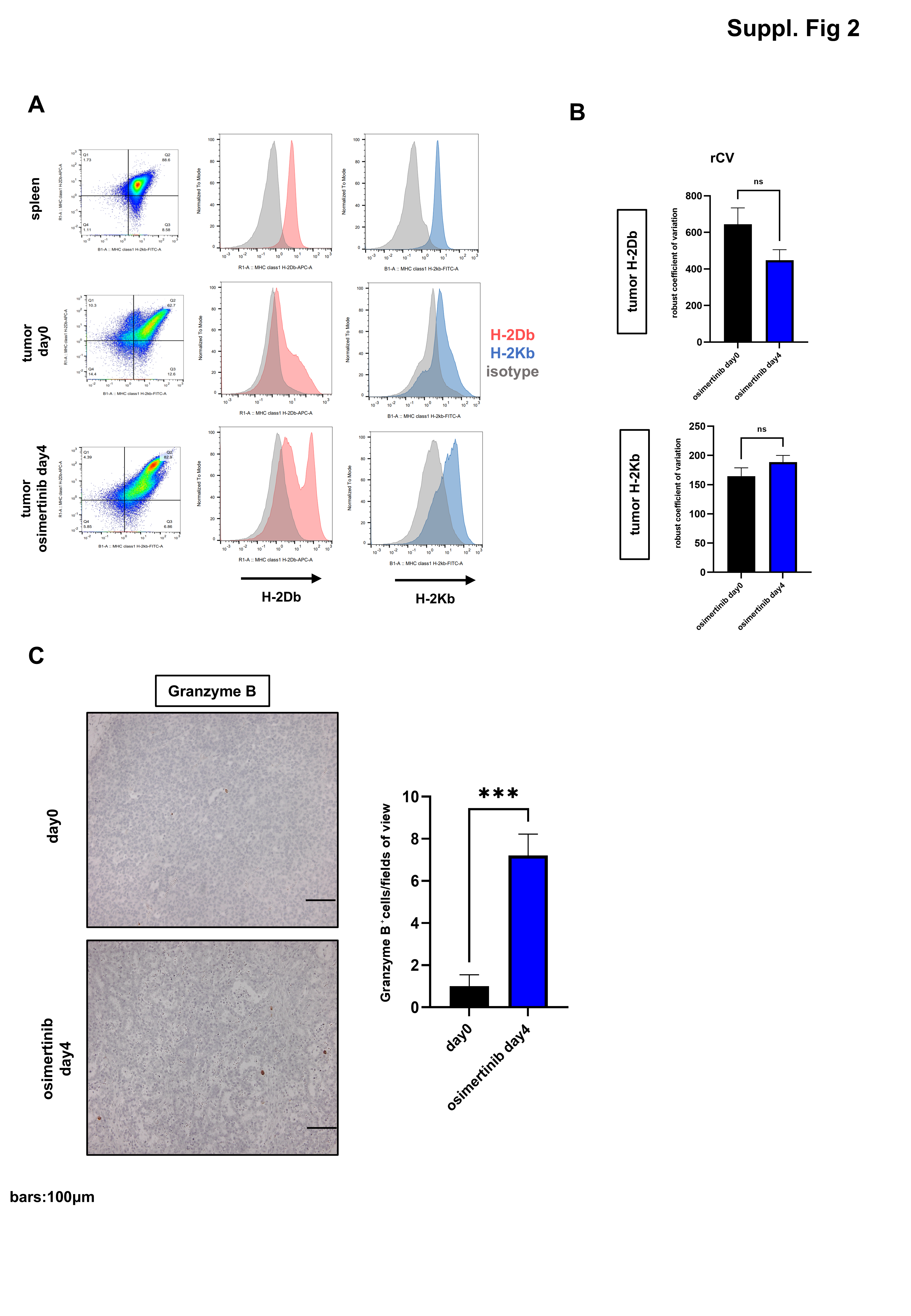

Supplement: Supplementary file 2 — Fig. S2. Functional relevance of EGFR‐TKI‐induced immune modulation. (A) Representative flow cytometry data showing the expression of MHC class I proteins H‐2Kb and H‐2Db within the spleen and dissociated tumor cells. (B) robust coefficient of variation (rCV) (Day 0: n = 12 per group, Day 4: n = 10 per group) for H‐2Db and H‐2Kb are shown. Error bars represent the standard error. ns = not significant, Student's t‐test. (C) Representative images of Granzyme B immunohistochemistry (IHC) staining on tumors from Egfr‐mutant mice treated with osimertinib (15 mg·kg−1·day−1, via oral gavage [p.o.]) after 0 or 4 days. The Granzyme B⁺ cells were quantified using imagej software. Error bars represent the standard error (n = 5 fields of view per group). Scale bars: 100 μm. ***P < 0.001, Student's t‐test. [file MOL2-9999-0-s003.pdf]

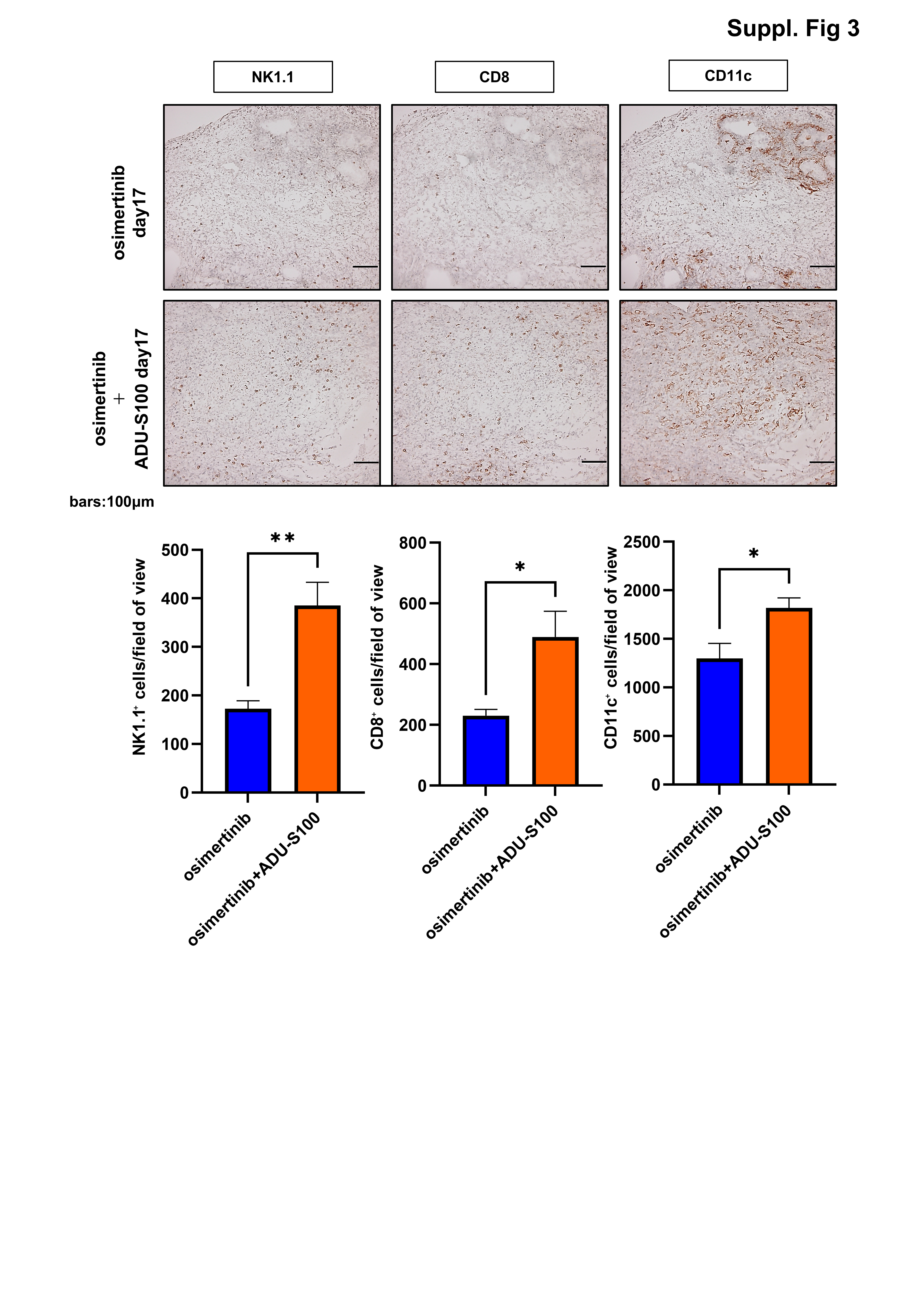

Supplement: Supplementary file 3 — Fig. S3. Immune cell infiltration in Egfr‐mutant lung tumors following osimertinib and ADU‐S100 treatment. Representative immunohistochemistry (IHC) staining images of NK1.1, CD8, and CD11c on Egfr‐mutant lung tumors from mice treated with osimertinib (15 mg·kg−1·day−1, via oral gavage [p.o.], 7 days/week) and combination of osimertinib (15 mg·kg−1·day−1, p.o., 7 days/week) and ADU‐S100 (50 μg, intratumorally, Day 1) for 14 days and after 3 days of drug withdrawal. The NK1.1⁺, CD8⁺, and CD11c⁺ cells were quantified using imagej software. Error bars represent the standard error (n = 5 fields of view per group). Scale bars: 100 μm. *P < 0.05; **P < 0.01, Student's t‐test. [file MOL2-9999-0-s005.pdf]

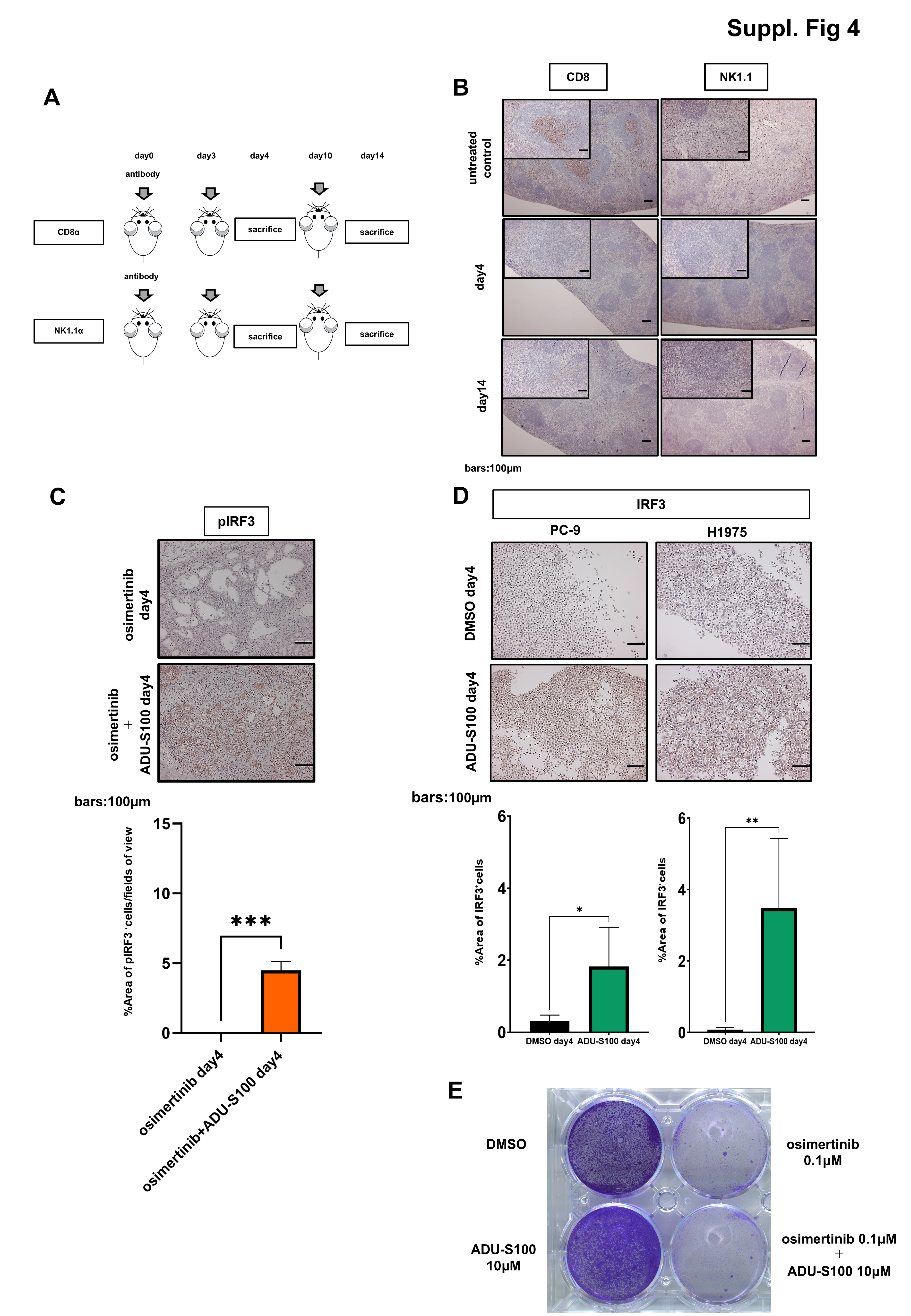

Supplement: Supplementary file 4 — Fig. S4. Efficiency of immune cell depletion and evaluation of the direct effects of ADU‐S100 in vitro. (A) Schematic image of the experimental schedule for immune cell depletion. (B) Representative immunohistochemistry (IHC) staining image of CD8 and NK1.1 in spleen tissues harvested on Day 4, Day 14 and untreated control. Scale bars: 100 μm. (C) Representative images of p‐IRF3 IHC staining on Egfr‐mutant lung cancer tumors from mice treated with osimertinib (15 mg·kg−1·day−1, via oral gavage [p.o.]) and combination of osimertinib (15 mg·kg−1·day−1, p.o.) and ADU‐S100 (50 μg, intratumoral administration, Day 1) for 4 days. %Area of p‐IRF3⁺ cells was quantified using imagej software. Error bars represent the standard error (n = 5 fields of view per group). Scale bars: 100 μm. ***P < 0.001, Student's t‐test. (D) Representative images of IRF3 IHC staining after 4 days of DMSO or ADU‐S100 (10 μmol·L−1) load. IRF3⁺ areas (%) were quantified using imagej software. Error bars represent the standard error (n = 5 fields of view per group). Scale bars: 100 μm. *P < 0.05, **P < 0.01, Student's t‐test. (E) Crystal violet assay of the murine Egfr‐mutant cell line (mDEL). Cancer cells were treated with 0.2% dimethyl sulfoxide, 0.1 μmol·L−1 osimertinib and/or 10 μmol·L−1 ADU‐S100 for 4 days. [file MOL2-9999-0-s001.pdf]

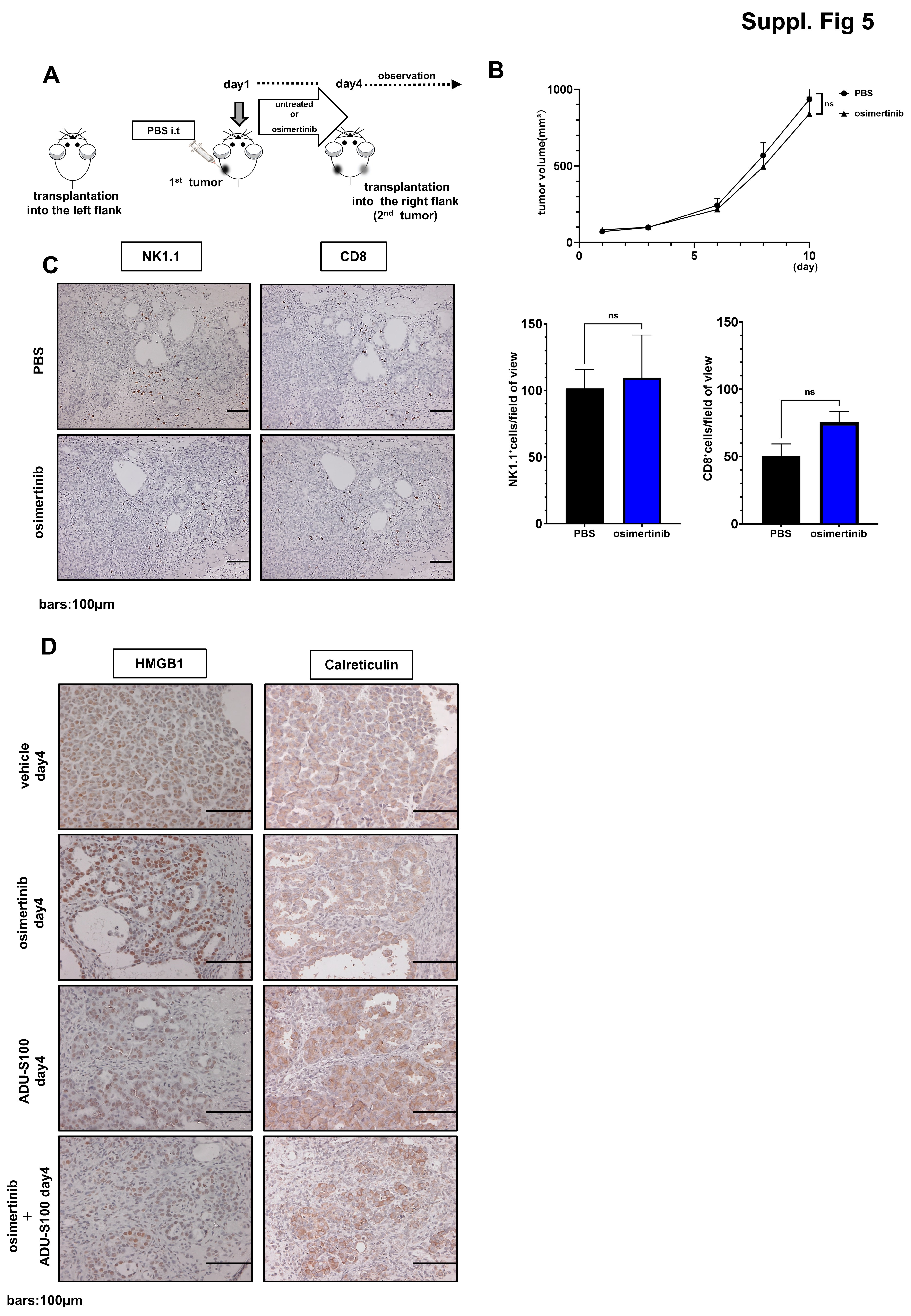

Supplement: Supplementary file 5 — Fig. S5. Absence of abscopal effect with osimertinib monotherapy and ADU‐S100‐induced immunogenic cell death in Egfr‐mutant lung tumors. (A) Schematic image of the treatment and transplantation schedule. (B) Secondary tumor growth. n = 4 tumors per group, 4 mice per group. Data shown are representative of two independent experiments with similar results. Error bars represent the standard error. ns = not significant, Student's t‐test. (C) Representative images of NK1.1 and CD8 immunohistochemistry (IHC) staining on secondary tumors 7 days after transplantation. The NK1.1⁺ and CD8⁺ cells were quantified using imagej software. Error bars represent the standard error (n = 5 fields of view per group). Scale bars: 100 μm. ns = not significant, Student's t‐test. (D) Representative images of HMGB1 and calreticulin (CRT) IHC staining on first tumors from mice treated with PBS (100 μL, intratumoral administration [i.t.], Day 1), osimertinib (15 mg·kg−1·day−1, oral gavage [p.o.]), ADU‐S100 (50 μg, i.t., Day 1), and combination of osimertinib (15 mg·kg−1·day−1, p.o.) and ADU‐S100 (50 μg, i.t., Day 1) for 4 days. Scale bars: 100 μm. [file MOL2-9999-0-s004.pdf]
